# Modeling of Cooperative Robotic Systems and Predictive Control Applied to Biped Robots and UAV-UGV Docking with Task Prioritization

**DOI:** 10.3390/s24103189

**Published:** 2024-05-17

**Authors:** Baris Taner, Kamesh Subbarao

**Affiliations:** Department of Mechanical and Aerospace Engineering, The University of Texas at Arlington, 500 W. First St., Arlington, TX 76019, USA

**Keywords:** cooperation, model predictive control, rover, quadcopter, docking

## Abstract

This paper studies a cooperative modeling framework to reduce the complexity in deriving the governing dynamical equations of complex systems composed of multiple bodies such as biped robots and unmanned aerial and ground vehicles. The approach also allows for an optimization-based trajectory generation for the complex system. This work also studies a fast–slow model predictive control strategy with task prioritization to perform docking maneuvers on cooperative systems. The method allows agents and a single agent to perform a docking maneuver. In addition, agents give different priorities to a specific subset of shared states. In this way, overall degrees of freedom to achieve the docking task are distributed among various subsets of the task space. The fast–slow model predictive control strategy uses non-linear and linear model predictive control formulations such that docking is handled as a non-linear problem until agents are close enough, where direct transcription is calculated using the Euler discretization method. During this phase, the trajectory generated is tracked with a linear model predictive controller and addresses the close proximity motion to complete docking. The trajectory generation and modeling is demonstrated on a biped robot, and the proposed MPC framework is illustrated in a case study, where a quadcopter docks on a non-holonomic rover using a leader–follower topology.

## 1. Introduction

Planning a trajectory for complex robots such as a biped is a complex task as the free-floating base of the robot is moved by the discontinuous contact forces acting on their feet. This propulsion method requires attention in planning the motion of the contact points and the contact forces while considering the dynamic effects on the robot [1]. In addition, a straightforward optimal control formulation results in intractable non-linear programs, as stated in the literature [2,3].

There are multiple causes of complexity in planning the trajectory of a floating base robot. First of all, the pose of the floating base is described as unactuated base coordinates with six degrees of freedom (DoF). Then, actuated joint coordinates of legs are added on top of that, which results in relatively large joint coordinates [1]. Therefore, the trajectory optimization of such a system should find optimal values for all these coordinates. Secondly, contact between the feet and the terrain must comply certain contact conditions such as unilateral contact forces [4]. In the literature, this problem is handled as a trajectory optimization problem using numerical optimization techniques such as direct optimal control methods [5], indirect optimal control methods [6], dynamic programming [7], and sequential methods [8]. In this way, trajectory of the robot, along with calculated joint coordinates, torques, and contact forces, are calculated by considering the upper-level constraints on the task.

The complexity of the problem is reduced in multiple ways. One way to carry this out is to use low-fidelity dynamic models for the robot such as single rigid body dynamics model, where lumped inertia is attached to the body frame and actuated links are assumed to be moving slowly and having low inertia [2]. Another way to reduce complexity is to split the the optimization into smaller sub-problems and to pre-define some portions of the trajectory such as footholds [9].

However, simplifications in robot model and optimization problems are compromised with the complexity of the motion that can be calculated with trajectory optimization. For this reason, having means to simplify full-body dynamics without compromising the fidelity of the model is vital. Recently, the trajectory optimization problem was distributed into smaller alternating sub-problems, where one first satisfies dynamic constraints of the problem by finding robot momentum and contact forces, and then one finds the leg kinematics that satisfy robot dynamics. This is denoted as centroidal and whole-body model splitting and was first introduced in the work [8], where a sequential optimal control formulation was represented. In another work based on the same splitting of whole-body motion and centroidal dynamics, the locomotion problem is cast into a mathematical framework based on Alternating Direction Method of Multipliers (ADMM) [10]. This paper exploits the natural splitting between centroidal and manipulator dynamics and ensures consensus between these models. Another recent example uses the same splitting and introduces an accelerated ADMM algorithm to solve the locomotion problem. [3].

Besides the centroidal and whole body splitting of the dynamic model of a legged robot, there is obviously another split between the portions of the body. This splitting is naturally defined by the design of legged robots and referred to as body and limbs. Depending on the design, they can contain a single body or a set of multi-bodies and interconnections between these sets maintained over a connection node, which is a joint. A formal way to describe the cooperation between these sets already exists in the literature within a cooperative system framework [11]. This framework defines a relationship between independent agents using communication graphs, which dictate a mapping to the information flow among agents. Cooperative system frameworks are used in many areas such as increasing performance of a sensory network in localization of data [12] or calculating the communication topology between dynamic agents within the cooperative system [13]. It is also used in designing controllers for agents within a network [14]. All the applications have one thing in common, which is the distributed modeling of the cooperative system and the distributed calculation of the variables to reach the desired objective.

Autonomous aerial systems have become essential in numerous practical fields, such as in public safety, as a surveillance tool and first response [15,16,17]. It has also been studied for the delivery of goods by the industry [18,19]. A critical task during the operation of an aerial system is docking maneuver, where it might be approaching a stationary or a moving platform [20,21] to drop/load cargo. In addition, aerial vehicles require refueling or recharging to extend their workspace [22]. Docking is an intricate maneuver that necessitates the awareness of the docking path’s constraints regarding flight safety and the docked platform’s attitude [23]. In addition, nonlinear effects such as the wake of the leading agent due to the proximity flight must be considered [24] as well as the ground effect [25]. Thus it is clear that the characteristic of this maneuver requires the agent to handle certain constraints and uncertainties.

A popular control method to handle previously described constraints and calculate control actions is model-predictive control (MPC). An MPC reaches the desired control action by minimizing a given objective using linear and non-linear optimization theory, as applied in the case of a tracking controller [26]. MPC also allows the integration of secondary tasks into decision-making, as in [27], which makes it a unified strategy to handle docking maneuvers without requiring ad-hoc integration of multiple frameworks that increase complexity. Implementing MPC-based docking strategies for space applications exists in case studies, where line of sight constraints are satisfied, energy-saving strategies are pursued, and docking on tumbling objects are executed [28,29,30].

Autonomous docking for aerial vehicles requires state information of the docked platform, which receives the docking agent. Therefore it certainly requires an on-board or external mechanism to sense and estimate states. On-board sensors such as cameras or LiDAR are widely implemented solutions (see [31]), yet they are bounded with range limitations. External mechanisms to obtain state information about the docked platform are sensors placed on the agents, except for the docking agent or external observers. As a result, this information is shared over a communication grid as illustrated in [32]. Besides state information, some of the autonomous docking literature can be grouped in terms of cooperation at the controller level. In one of the works, a central controller calculates lateral and longitudinal velocity commands for both of the docking maneuver devices: an uncrewed ground vehicle (UGV) and an uncrewed aerial vehicle (UAV). Then, these control signals are fed into these agents [33]. In the extension of this work, an MPC strategy is utilized on the same system, where both agents are cooperatively trying to execute docking maneuvers [34]. In another work, a multirotor docks onto a fixed-wing platform, where only the multirotor executes the docking maneuver [21]. The aerial refueling problem is addressed as a docking problem in [22]. However, only the docking platform and the boom are manipulated. Recent work studies an uncrewed sea surface vehicle (USV) landing and designs an MPC controller for a multirotor, where cooperative docking is executed [35]. However, the docking trajectory calculation is calculated on the multirotor and shared back to the USV.

Motivation

In this context, this paper aims to keep the model fidelity of the biped robot higher than a single body dynamics and adopt a cooperative system framework in defining dynamics of the robot. As a result, the robot will be defined by multiple cooperating agents, where cooperation is introduced to the trajectory optimization by an adjacency matrix as a constraint. In this way, lumped trajectory optimization is converted to a separated objectives and constraints that are linearly related.

When multiple agents are communicating with each other and obeying specific rules, i.e., collision avoidance, velocity matching, and staying within the vicinity of the neighbors, the aggregate of these agents are called cooperative agents [36] and the application of these systems are vast due to the advantages. Popular applications include uncrewed vehicles [37] and space applications [38]. The nature of autonomous docking makes the system performing this maneuver a cooperative system. However, it is not formally addressed as such in the vast majority of the literature. This is due to the fact that in most scenarios, one of the vehicles is assumed to be tightly controlled and hence passive. The control laws are then derived for the remaining active vehicle. On the other hand, controllers cooperatively addressing this problem are mainly centralized on a ground controller or in one of the agents, which could suffer in performance or even fail due to uncertainties in the communication. Apart from the centralized implementation of cooperative docking, there are a few applications where both agents take part in the docking maneuver. Since the cooperative control framework allows various communication topologies, methods based on MPC with local neighbor state information can be applied. Besides the cooperative aspect, a prioritization of the states to track during docking is typically not studied in the literature. As summarized before, agents that are too far apart from each other first could first minimize the positional difference, which is carried out to be in a feasible solution set when the MPC for docking is initiated. Instead of handling this problem as two separate sub-problems, an automated prioritization of tracking certain states can be defined so that linear states can be given higher priority over the angular states (see [39]).

Contributions

The key contributions of this paper are as follows. The methods introduced in this paper are used to divide the lumped multi-body model of a large robot-system into cooperative multi-bodies and address the trajectory optimization problem using this distributed model. In the case of the biped robot, although it has light weight legs compared to the shoulder and the floating base, the biped robot will be modeled as three cooperative agents, which are the floating base, right shoulder, and left shoulder. All agents are defined as multi-bodies, as defined in the following sections. The contributions of this paper are summarized as follows:

- This method divides the EoM of the biped robot into smaller cooperative agents, which has simpler EoMs. Agents with simpler dynamics result in simpler equality constraints for the trajectory optimization.
- The non-linear programming formulation given in this paper cast the trajectory optimization problem with single objective and single augmented Hamiltonian into split objectives and constraints.
- This paper also proposes a cooperative control strategy based on MPC for docking. The designed strategy implements a non-linear and a linear MPC for the coarse approach (long distance) and the delicate docking maneuver (short distance) based on the same objective function with tailored optimization strategies. A leader–follower type of topology is adopted, where the quadcopter docks on the UGV. As a showcase, this controller performs short- and long-distance docking of a quadcopter on a UGV.
- Formulation of the MPC includes task prioritization, which is based on a null-space projection of the tasks being ranked. The formulation is adopted from [40] by defining the docking task in terms of the docking agents’ Degrees of Freedom (DoF).

The organization of the paper is such that Section 2 expresses governing equations of agents and the underlying graph structure. Following that, Section 8 provides a task-prioritization and MPC strategy for docking. The simulation results are shared in Section 9. Finally, conclusions are provided in Section 11.

## 2. Notation and Preliminaries

The notation of this work is as follows. s∈R, $v\in R^{n_{v}}$ and $M\in R^{n_{r}\times n_{c}}$ represent the arbitrary scalar, vector, and matrix. $F_{I}$ and $F_{B}$ represent the inertial and body frames, respectively, where the expression of a vector for these frames is written as v  i ,i=I,B. Unit vectors in orthonormal frames are denoted as $u_{1}={\left[\begin{array}{ccc} 1 & 0 & 0 \end{array}\right]}^{T}$, $u_{2}={\left[\begin{array}{ccc} 0 & 1 & 0 \end{array}\right]}^{T}$ and $u_{3}={\left[\begin{array}{ccc} 0 & 0 & 1 \end{array}\right]}^{T}$. The composite rotation matrix CB I ∈SO(3) is written from $F_{B}$ to $F_{I}$, the rotation sequence is depicted as z−y−x, and associated Euler angles are denoted as ψ, θ and ϕ. g=9.81 m/s^2^ is the gravitational acceleration. $col{\left\{\cdot \right\}}_{m=1}^{M}$, $row{\left\{\cdot \right\}}_{l=1}^{M}$, and $diag{\left\{\cdot \right\}}_{m=1}^{M}$ represent column, row and diagonal concatenation, respectively, of the entity within the parenthesis.

## 3. Underlying Graph Structure

The communication among agents in the cooperative systems are described by the graph G=(N,E), which consists of node set N and edge set E [41]. Edge set E⊂N×N is given between nodes i∈N and j∈N such that (j,i)∈E denotes node *i* receives information from *j*. Let $n_{w}$ be an arbitrary signal dimension, and then the adjacency matrix; shared signal sizes among agents are assumed to be identical and equal to $n_{w}$. $A=\left[a_{ij}\right]⊗I_{n_{w}}\in R^{N\cdot n_{w}\times N\cdot n_{w}}$ of G is composed of weighting scalars $a_{ij}$, where $a_{ij}$ quantifies the strength of the connection from node *j* to node *i*. *N* is the number of agents in the cooperative system (CS). Formally, $A_{ij}$ is described by the following equation.

(1) $$A_{ij}=\left\{\begin{array}{cc} a_{ij}>0, & j\neq i,\;\;(j,i)\in E\\ a_{ij}=0, & otherwise \end{array}\right.$$

## 4. ASLB—A Bipedal Robot for Dynamics Locomotion

### 4.1. ASLB System Composition

**Figure 1:**
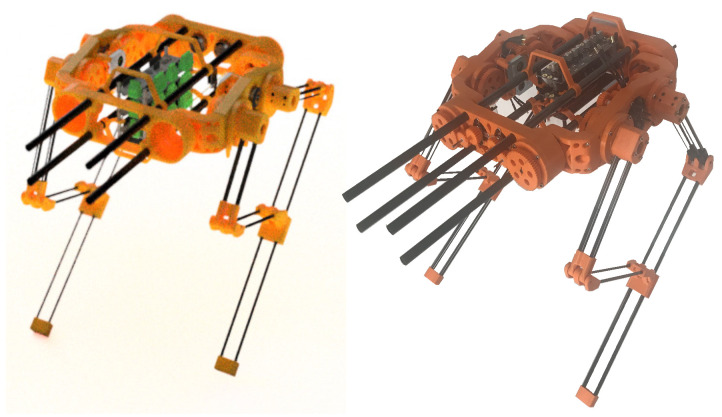
Three-dimensional model (**left**) and manufactured prototype of ASLB (**right**).

ASLB is a floating bipedal platform in which each leg is composed of hybrid structure with three degrees of freedom (DoFs). Specifically, starting from the body, the kinematic structure of legs are a revolutionjoint (at shoulder) followed by a parallel 5R mechanism. This kinematics result in three actuated and two passive joint coordinates in each leg. A rendered 3D model and manufactured prototype of ASLB is provided in Figure 1.

Table 1 provides a summary of the key variables and terms used for the development of the ASLB dynamic model.

### 4.2. ASLB Kinematics

**Figure 2:**
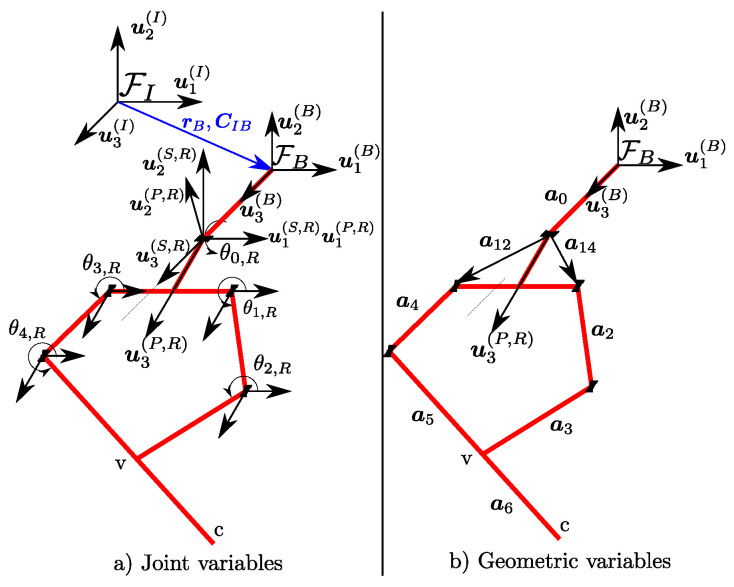
Kinematic structure of ASLB.

Kinematic model of the floating platform starts with a set of unactuated joints that gives six DoFs to the mobile platform. These joints are collected in a column matrix $q_{B}\in R^{3}\times SO\left(3\right)$. Starting from the inertial frame, joints are located in a sequence such that first translational joints $q_{t,B}\;=\;{\left[q_{1},q_{2},q_{3}\right]}^{T}\in R^{3}$ are located in respective *x*, *y* and *z* directions. Assuming the euler sequence of YZX, rotational joints are $q_{r,B}\;=\;{\left[q_{4},q_{5},q_{6}\right]}^{T}\in R^{3}$. Body frame $F_{B}$ is kinematically represented with respect to $F_{I}$ by $q_{B}\;=\;{\left[q_{t,B}^{T},\;q_{r,B}^{T}\right]}^{T}$ such that $r_{B}\left(q_{t,B}\right)$ and $C_{IB}\left(q_{r,B}\right)$ are translation and rotation matrices from $F_{I}$ to $F_{B}$, respectively. There are two legs attached to the body, and respective joints are represented by $\theta_{i}\in R^{n_{i}}$, where $n_{i}=5$ and i=R,L. In total, the combined DoF for legs are given as $n_{a}\;=\;10$. The composition of $\theta_{i}$ for each leg is given as $\theta_{i}\;=\;{\left[\theta_{0,i}\;\theta_{1,i}\;\theta_{2,i}\;\theta_{3,i}\;\theta_{4,i}\right]}^{T}$, where there are three active and two passive joints, respectively $\theta_{a,i}\;=\;{\left[\theta_{0,i}\;\theta_{1,i}\;\theta_{3,i}\right]}^{T}\in R^{3}$ and $\theta_{p,i}\left(\theta_{a,i}\right)\;=\;{\left[\theta_{2,i}\left(\theta_{a,i}\right)\;\theta_{4,i}\left(\theta_{a,i}\right)\right]}^{T}$. Passive joints can be written as a function of $\theta_{a,i}$ based on a velocity constraint as described in Section 4.2.1. As a result, total joint space of ASLB is given by $q\;=\;{\left[q_{B},\;\theta_{a,R},\;\theta_{a,L}\right]}^{T}\in R^{12}$. The kinematic structure of the right leg is illustrated in Figure 2.

#### 4.2.1. Passive—Active Joint Relation

As described in [42] starting from the origin of the body frame $F_{B}$, there are two chains to reach point v on both legs. Let $R_{*}$ represent elementary rotation matrix, where ∗=x,y,z are active axes. Then these two chains can be written as in Equation (2).

(2) $$\begin{array}{c} r_{v,1}=a_{12}+R_{z}\left(\theta_{1,R}\right)a_{2}+R_{z}\left(\theta_{1,R}+\theta_{2,R}\right)a_{2}\\ r_{v,2}=a_{14}+R_{z}\left(\theta_{3,R}\right)a_{2}+R_{z}\left(\theta_{3,R}+\theta_{4,R}\right)a_{2} \end{array}$$

Differentiating Equation (2) results in velocity equations of $v_{r,1}$ and $v_{r,2}$, which are equal. Writing this relationship as below relates the passive joints to active joints as described in Equation (3). Let ${\bar{\theta }}_{a,i}\;=\;{\left[\theta_{1,i}\;\theta_{3,i}\right]}^{T}$ denote the set of active joints related to the closed loop, then $J_{a,i}\in R^{2}$ becomes a square matrix.

(3) $$\begin{array}{cc} v_{r,1}\;=\; & J_{r,1}{\left[\begin{array}{cc} {\bar{\theta }}_{a,i}^{T} & \theta_{p,i}^{T} \end{array}\right]}^{T}\\ v_{r,2}\;=\; & J_{r,2}{\left[\begin{array}{cc} {\bar{\theta }}_{a,i}^{T} & \theta_{p,i}^{T} \end{array}\right]}^{T}\\ 0\;=\; & \left(J_{r,1}-J_{r,2}\right){\left[\begin{array}{cc} {\bar{\theta }}_{a,i}^{T} & \theta_{p,i}^{T} \end{array}\right]}^{T}\\ \;=\; & \left[\begin{array}{cc} J_{a} & J_{p} \end{array}\right]{\left[\begin{array}{cc} {\bar{\theta }}_{a,i}^{T} & \theta_{p,i}^{T} \end{array}\right]}^{T} \end{array}$$

Finally, passive joints are related to active joints as given in Equation (4).

(4) $$\begin{array}{c} \theta_{p,i}\;=\;J_{pa}{\bar{\theta }}_{a,i}\\ J_{pa}\;=\;-J_{p,i}^{-1}J_{a,i} \end{array}$$

#### 4.2.2. Forward Kinematics

Forward kinematics for each leg are used to calculate the position of the contact point *c* with respect to origin of $F_{B}$, as illustrated in Figure 2. The aforementioned position vector is denoted as $r_{c}$. As mentioned in Section 4.2.1 the active and passive joint angles are related to each other and unless the passive joints are measured by sensors, they have to be calculated from this relationship. To do so, position vectors $r_{v,1}$ and and $r_{v,2}$ are arranged as shown below.   

(5) $$\begin{array}{cc} \; & \left[\begin{array}{c} \cos(\theta_{3,R}+\theta_{4,R})\\ \sin(\theta_{3,R}+\theta_{4,R})\\ 0 \end{array}\right]a_{5,x}=\\ \; & -\left[\begin{array}{c} \cos(\theta_{1,R}+\theta_{2,R})\\ \sin(\theta_{1,R}+\theta_{2,R})\\ 0 \end{array}\right]a_{3,x}+\left[\begin{array}{c} a_{12,x}-a_{14,x}\\ a_{12,y}-a_{14,y}\\ a_{12,z}-a_{14,z} \end{array}\right]\\ \; & +\left[\begin{array}{c} \cos(\theta_{1,R})\\ \sin(\theta_{1,R})\\ 0 \end{array}\right]a_{2,x}-\left[\begin{array}{c} \cos(\theta_{3,R})\\ \sin(\theta_{3,R})\\ 0 \end{array}\right]a_{4,x} \end{array}$$

The first two rows of Equation (5) can be written in a minimal form as provided in Equation (6) such that terms including $\theta_{3,R}$, $\theta_{4,R}$ are left alone.

(6) $$\begin{array}{cc} \; & \left[\begin{array}{c} \cos(\theta_{3,R}+\theta_{4,R})\\ \sin(\theta_{3,R}+\theta_{4,R}) \end{array}\right]\;=\;\left[\begin{array}{c} T_{x}\\ T_{y} \end{array}\right]+\frac{a_{3,x}}{a_{5,x}}\left[\begin{array}{c} \cos(\theta_{1,R}+\theta_{2,R})\\ \sin(\theta_{1,R}+\theta_{2,R}) \end{array}\right]\\ \; & T_{x}\;=\;\frac{1}{a_{5,x}}\left(a_{12,x}-a_{14,x}+\cos\left(\theta_{1,R}\right)+\cos\left(\theta_{3,R}\right)\right)\\ \; & T_{y}\;=\;\frac{1}{a_{5,x}}\left(a_{12,y}-a_{14,y}+\sin\left(\theta_{1,R}\right)+\sin\left(\theta_{3,R}\right)\right) \end{array}$$

Elements of Equation (6) are squared and summed to obtain Equation (7). Then trigonometric expressions are written in terms of tangents of the half angles, which leads to the a solution to $\theta_{12,R}=\theta_{1,R}+\theta_{2,R}$ as provided in Equation (8).

(7) $$\begin{array}{c} T_{x}^{2}+T_{y}^{2}+{\frac{a_{3,x}}{a_{5,x}}}^{2}+2T_{x}\frac{a_{3,x}}{a_{5,x}}\cos\left(\theta_{12,R}\right)+2B\sin\left(\theta_{12,R}\right)=1 \end{array}$$

(8) $$\begin{array}{cc} \theta_{12,R}\; & =\;2\tan^{-1}\left(T_{12}\right)\\ T_{12}\; & =\;\frac{-2C_{3}\pm \sqrt{{\left(2C_{3}\right)}^{2}-4\left(C_{1}-C_{2}\right)\left(C_{1}+C_{2}\right)}}{2(C_{1}-C_{2})}\\ C_{1}\; & =\;T_{x}^{2}+T_{y}^{2}+{\frac{a_{3,x}}{a_{5,x}}}^{2}-1\\ C_{2}\; & =\;2T_{x}\frac{a_{3,x}}{a_{5,x}}\\ C_{3}\; & =\;2T_{y}\frac{a_{3,x}}{a_{5,x}} \end{array}$$

Using $\theta_{12,R}$, one can write Equation (9) and solve for $\theta_{34,R}=\theta_{3,R}+\theta_{4,R}$. Finally, tip point location $r_{c}$ is calculated as provided in Equation (10).

(9) $$\begin{array}{cc} \theta_{34,R}\; & =\;atan2(s_{34,R},c_{34,R})\\ \left[\begin{array}{c} c_{34,R}\\ s_{34,R} \end{array}\right]\; & =\;\left[\begin{array}{c} T_{x}+\frac{a_{3,x}}{a_{5,x}}\cos\left(\theta_{12,R}\right)\\ T_{y}+\frac{a_{3,x}}{a_{5,x}}\sin\left(\theta_{12,R}\right) \end{array}\right] \end{array}$$

(10) $$\begin{array}{cc} r_{c}\;=\;a_{0}+R_{x}\left(\theta_{0,R}\right)(a_{14} & +R_{z}\left(\theta_{3,R}\right)a_{4}\\ & +R_{z}\left(\theta_{34,R}\right)\left(a_{5}+a_{6}\right)) \end{array}$$

#### 4.2.3. Inverse Kinematics

**Figure 3:**
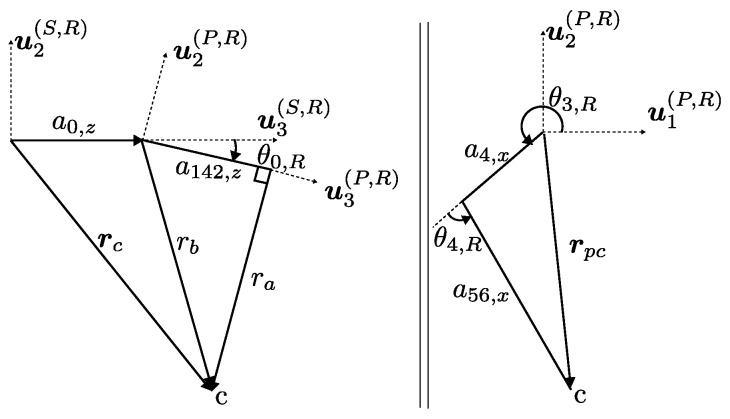
Geometric entities related to calculation of $\theta_{0,R}$.

Inverse kinematics is illustrated on right leg, and the calculations provided here can be duplicated for left leg. Inverse kinematics solutions in this work rely on the geometric calculation of $\theta_{0,R}$ as provided in Equation (11). The geometric entities are illustrated in Figure 3.

(11) $$\begin{array}{cc} \theta_{0,R}\; & =\;atan2(\sin\left(\theta_{0,R}\right),\cos\left(\theta_{0,R}\right))\\ \left[\begin{array}{c} \cos(\theta_{0,R})\\ \sin(\theta_{0,R}) \end{array}\right]\; & =\;{\left[\begin{array}{cc} a_{142,z} & -r_{a}\\ -r_{a} & -a_{142,z} \end{array}\right]}^{-1}\left[\begin{array}{c} r_{z}-a_{0,z}\\ r_{y}-a_{0,y} \end{array}\right]\\ r_{1}\; & =\;r-a_{0}\\ r_{a}\; & =\;\sqrt{r_{1,y}^{2}+r_{1,z}^{2}-a_{14,z}^{2}}\\ a_{142,z}\; & =\;a_{14,z}+a_{2,z} \end{array}$$

To calculate active joints $\theta_{1,R}$ and $\theta_{3,R}$x and y components of the position vector $r_{pc}$ as given in Equation (12) are expanded in Equation (13).

(12) $$\begin{array}{cc} r_{pc,x}\; & =\;a_{4,x}\cos\left(\theta_{3,R}\right)+a_{56,x}\cos\left(\theta_{3,R}+\theta_{4,R}\right)\\ r_{pc,y}\; & =\;a_{4,x}\sin\left(\theta_{3,R}\right)+a_{56,x}\sin\left(\theta_{3,R}+\theta_{4,R}\right) \end{array}$$

(13) $$\begin{array}{cc} r_{pc,x}\; & =\;a_{4,x}\cos\left(\theta_{3,R}\right)+a_{56,x}\left(\cos\left(\theta_{3,R}\right)\cos\left(\theta_{4,R}\right)-\sin\left(\theta_{3,R}\right)\sin\left(\theta_{4,R}\right)\right)\\ r_{pc,y}\; & =\;a_{4,x}\sin\left(\theta_{3,R}\right)+a_{56,x}\left(\cos\left(\theta_{3,R}\right)\sin\left(\theta_{4,R}\right)+\sin\left(\theta_{3,R}\right)\cos\left(\theta_{4,R}\right)\right) \end{array}$$

Equation (13) is rewritten as in Equation (14), and each scalar equation can be squared and summed as in Equation (15).

(14) $$\begin{array}{cc} \cos(\theta_{3,R})\; & =\;\left(r_{pc,x}/a_{4,x}\right)+\left(a_{56,x}/a_{4,x}\right)\cos\left(\theta_{3,R}+\theta_{4,R}\right)\\ \; & =\;C_{4}+C_{5}\cos\left(\theta_{3,R}+\theta_{4,R}\right)\\ \sin(\theta_{3,R})\; & =\;\left(r_{pc,y}/a_{4,x}\right)+\left(a_{56,x}/a_{4,x}\right)(\sin\left(\theta_{3,R}+\theta_{4,R}\right)\\ \; & =\;C_{6}+C_{5}\sin\left(\theta_{3,R}+\theta_{4,R}\right) \end{array}$$

(15) $$\begin{array}{cc} 1\; & =\;C_{4}^{2}+C_{6}^{2}+C_{5}^{2}+2C_{4}C_{5}\cos\left(\theta_{3,R}+\theta_{4,R}\right)+2C_{6}C_{5}\sin\left(\theta_{3,R}+\theta_{4,R}\right)\\ 0\; & =\;\left(C_{4}^{2}+C_{6}^{2}+C_{5}^{2}-1\right)+\left(2C_{4}C_{5}\right)\cos\left(\theta_{3,R}+\theta_{4,R}\right)+\left(2C_{6}C_{5}\right)\sin\left(\theta_{3,R}+\theta_{4,R}\right)\\ 0\; & =\;C_{7}+C_{8}\cos\left(\theta_{3,R}+\theta_{4,R}\right)+C_{9}\sin\left(\theta_{3,R}+\theta_{4,R}\right) \end{array}$$

Then, using tangents of the half angle, Equation (15) can be further manipulated in Equation (16). The solution to $\theta_{34,R}$ can be calculated by solving the quadratic problem for $T_{34}$ as illustrated in Equation (8).

(16) $$\begin{array}{c} 0\;=\;\left(C_{7}-C_{8}\right)T_{34}^{2}+2C_{9}T_{34}+\left(C_{7}+C_{8}\right) \end{array}$$

After finding the solution to $\theta_{34,R}$, these values are inserted into Equation (12) to calculate $\theta_{3,R}$. This completes the solution to one chain of the leg mechanism.

**Figure 4:**
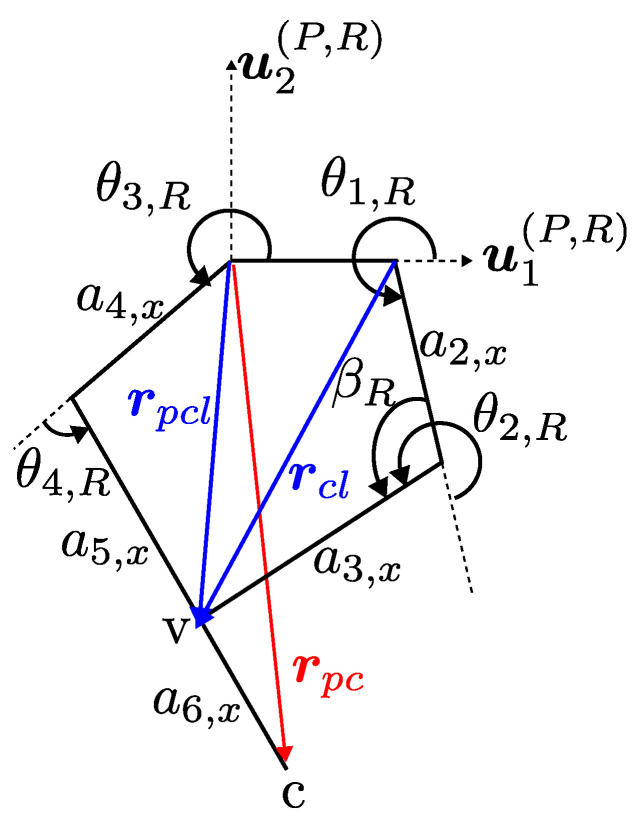
Geometric entities related to calculation of $\theta_{1,R}$.

Geometric definitions such as $r_{pcl}$, $r_{cl}$ are provided in Figure 4 to calculate the joint variables on the other chain that contains $a_{2}$ and $a_{3}$.

Using the known joint variables, point *v* is represented from origin of the joint 3 with a vector denoted as $r_{pcl}$. Alternatively, point *v* can also be represented from the origin of the joint 1 with a vector that is $r_{cl}$. These vectors are defined on (P,R) plane, where the 5R mechanism lies, and provided in Equations (17) and (18), respectively.

(17) $$\begin{array}{c} r_{pcl,x}\;=\;r_{pc,x}-a_{6,x}\cos\left(\theta_{3,R}+\theta_{4,R}\right)\\ r_{pcl,y}\;=\;r_{pc,y}-a_{6,x}\sin\left(\theta_{3,R}+\theta_{4,R}\right) \end{array}$$

(18) $$\begin{array}{cc} r_{cl,x}\; & =\;a_{2,x}\cos\left(\theta_{1,R}\right)+a_{3,x}\cos\left(\theta_{1,R}+\theta_{2,R}\right)\\ r_{cl,y}\; & =\;a_{2,x}\sin\left(\theta_{1,R}\right)+a_{3,x}\sin\left(\theta_{1,R}+\theta_{2,R}\right) \end{array}$$

Scalar equations $r_{cl,x}$ and $r_{cl,y}$ of Equation (18) are squared, summed, and reorganized as provided in Equation (19) to calculate $\beta_{R}$, which leads to $\theta_{2,R}$.

(19) $$\begin{array}{cc} \cos(\beta_{R})\; & =\;-\left(\frac{r_{cl,x}^{2}+r_{cl,y}^{2}-a_{2,x}^{2}-a_{3,x}^{2}}{2a_{2,x}a_{3,x}}\right)\\ \beta_{R}\; & =\;acos\left(-\frac{r_{cl,x}^{2}+r_{cl,y}^{2}-a_{2,x}^{2}-a_{3,x}^{2}}{2a_{2,x}a_{3,x}}\right)\\ \theta_{2,R}\; & =\;\pi +\beta_{R} \end{array}$$

To calculate $\theta_{1,R}$, $r_{cl,x}$ and $r_{cl,y}$ are represented as a function of $r_{pcl}$. This is given in Equation (20) in matrix form, where terms with $\theta_{1,R}$ are the unknown variables. The solution to Equation (20) for $\theta_{1,R}$ finishes the inverse kinematic solution of the right leg. The procedure for the left leg is identical.

(20) $$\begin{array}{c} \left[\begin{array}{c} r_{pcl,x}+\left(a_{14,x}-a_{12,x}\right)\\ r_{pcl,y} \end{array}\right]\;=\;\\ \left[\begin{array}{cc} a_{2,x}+a_{3,x}\cos\left(\theta_{2,R}\right) & -a_{3,x}\sin\left(\theta_{2,R}\right)\\ a_{3,x}\sin\left(\theta_{2,R}\right) & a_{2,x}+a_{3,x}\cos\left(\theta_{2,R}\right) \end{array}\right]\left[\begin{array}{c} \cos(\theta_{1})\\ \sin(\theta_{1}) \end{array}\right]\\ \theta_{1,R}\;=\;atan2\left(\sin\left(\theta_{1}\right),\cos\left(\theta_{1}\right)\right) \end{array}$$

### 4.3. ASLB Dynamics

Based on the generalized coordinates, multi-body dynamics of ASLB are formulated as in (Equation (21))

(21) $$\begin{array}{c} M\left(q\right)\ddot{q}+C\left(q,\dot{q}\right)+G\left(q\right)=S\tau +J_{C,i}^{T}F_{C,i} \end{array}$$

where $M\left(q\right)\in R^{12\times 12}$, $C\left(q,\dot{q}\right)\in R^{12}$, and $G\left(q\right)\in R^{12}$ are the generalized mass, Coriolis and centrifugal, and gravitational terms, respectively. $S\in R^{12\times 6}$, $\tau \in R^{6}$, $F_{C,i}\in R^{3}$ and $J_{C,i}\in R^{3\times 12}$ are the selection matrix for the actuated joints of respective legs, actuated joint torques, geometric Jacobian of the tip point of the $i^{th}$ leg, and external force on the tip of $i^{th}$ leg.

Let $x\;=\;{\left[q,\;\dot{q}\right]}^{T}\in R^{24}$, $u_{\tau }\;=\;\tau$, and $u_{c}\;=\;F_{C,i}\;\forall i$ be the states, inputs to motors and external forces acting on tip point of the legs, respectively; then, the non-linear dynamics of the robot can be written as in Equation (22).

(22) $$\begin{array}{cc} \dot{x} & \;=\;f(x,u_{\tau },u)\\ f(x,u_{\tau },u) & \;=\;\left[\begin{array}{c} \dot{q}\\ M{\left(q\right)}^{-1}\Theta \end{array}\right]\\ \theta & \;=\;-C\left(q,\dot{q}\right)-G\left(q\right)+S\tau +\sum_{i}J_{C,i}^{T}F_{C,i} \end{array}$$

The application of the graph-theoretic modeling for the ASLB by separating the legs, which are defined as **Agent 2** and **Agent 3**, from the floating base introduces simplifications to the overall complexity of the model and optimization. One of the simplifications appears in modeling as leg dynamics does not necessarily need to be modeled with respect to $F_{I}$ using $q_{B}$ but rather is better defined with respect to the $F_{B}$. This local representation of leg dynamics leads to following outcomes for the dynamics of the agents:

- The leg is only used to find adjacency and contact forces on the floating base and ground,
- The state space representation of the leg dynamics can be kept at velocity level.

Note that the contact forces and geometric properties of contact point must be converted to $F_{B}$.

**Figure 5:**
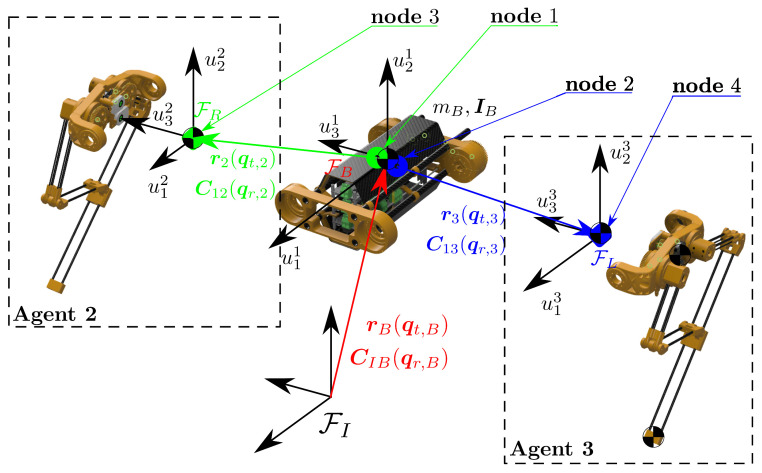
Cooperative interconnection between agents and generalized coordinates at every agent.

For ASLB, the floating base of the robot is denoted as Agent1, the right leg is denoted as Agent2, and the left leg is denoted as Agent3. Agent1 is defined with two nodes, node1 and node2; Agent2 is defined with node3; and Agent3 is defined with node4. The locations of these nodes are illustrated in Figure 5, and it should be noted that node1 and node2 coincide. Without any interconnection constraint, agents are independent of each other; however, there are rigid joints connecting them. In this case there are two bi-directional edges and these are (node3,node1)∈E and (node2,node4)∈E. Connections between nodes are assumed to be rigid, and then the adjacency matrix is composed of edge weights $a_{mn}=1$. This yields an adjacency matrix as in (23).

(23) $$A\;=\;\left[\begin{array}{cccc} 0 & 0 & 1 & 0\\ 0 & 0 & 0 & 1\\ 1 & 0 & 0 & 0\\ 0 & 1 & 0 & 0 \end{array}\right]$$

Finally, the relationships between cooperative agents are given in (24) by the Laplacian matrix. $I_{p}$ represents identity matrix with size *p*.

(24) $$L\;=\;I_{4}-A$$

Let $W\in R^{p}$ be the signal that is being shared between agents; then, L can be expanded as in (25) to comply with the signal dimension. ⊗ is the Kronecker product operator.

(25) $$L\;=\;L⊗I_{p}$$

Recalling that node1 and node2 are coincident, one can define the following adjacency constraints using the extended Laplacian definition given in (25)   

(26) $$\begin{array}{cc} \; & L_{x}\;=\;L\left[\begin{array}{c} x_{1}\\ x_{1}\\ x_{2}\\ x_{3} \end{array}\right]\;=\;0\\ \; & L_{w}\;=\;L\left[\begin{array}{c} W_{A,1}\\ W_{A,2}\\ W_{A,3}\\ W_{A,4} \end{array}\right]\;=\;0 \end{array}$$

Another aspect of splitting a lumped multi-body model into distributed cooperating multi-body models is generalized coordinates. This operation necessarily duplicates the generalized coordinates of the floating base in a lumped model to the distributed models. In addition to that, Agent2 and Agent3 have actuated joints of $q_{a,i}\;,\;i=2,3$. Therefore, generalized coordinates of the agents are defined as $q_{1}\;=\;{\left[q_{t,1}^{T}\;q_{r,1}^{T}\right]}^{T}\in R^{6}$, $q_{2}\;=\;{\left[q_{t,2}^{T}\;q_{r,2}^{T}\;q_{a,2}^{T}\right]}^{T}\in R^{9}$ and $q_{3}\;=\;{\left[q_{t,3}^{T}\;q_{r,3}^{T}\;q_{a,3}^{T}\right]}^{T}\in R^{9}$, respectively, for Agent1, Agent2, and Agent3. This is illustrated in Figure 5.

### 4.4. Agent Kinematics

The YZX Euler sequence is used in defining the composite rotations $C_{Ii}\left(q_{r,i}\right)$ from $F_{I}$ to $F_{i}$, where subscript *i* is the agent index and $F_{i}$ is the local origin of the agent. The contact point position and velocity of Agent2 and Agent3 are calculated with respect to $F_{B}$, which is denoted as in (27). $K^{+}\left(q_{i}\right)$ represents forward kinematics of leg *i* in Equation (27), which is explained in Section 4.2.

(27) $$\begin{array}{c} r_{c,i}\left(q_{i}\right)\;=\;K^{+}\left(q_{i}\right)\\ {\dot{r}}_{c,i}\left(q_{i},{\dot{q}}_{i}\right)\;=\;J_{c,i}{\dot{q}}_{i} \end{array}$$

### 4.5. Agent Dynamics

The dynamics of agents yield a similar equation as given in (22), and, for brevity, a representative Equation of Motion (EoM) is given in this section.

Generalized velocities are assigned to states of each agent as $x_{i}\;=\;{\dot{q}}_{i}$. External forces in distributed notation are divided into two, where the first one is denoted as $F_{C,i}$ and acts on the agents as a result of ground contact. The second external force is denoted as $F_{A,m}$ and exerted on the agents from the adjacent nodes. Adjacent nodes also transmit moment, $M_{A,m}$; therefore it is convenient to collect forces and moments at adjacent nodes such as a wrench, denoted as $W_{A,m}\;=\;{\left[F_{A,m}^{T}\;\;M_{A,m}^{T}\right]}^{T}$. As a result, the non-linear dynamics of each agent are written as given in (28). Let $x_{i}\;=\;\dot{q}\in R^{n_{xi}}$, $u_{\tau ,i}\;=\;\tau \in R^{n_{ti}}$, $u_{c,i}\;=\;F_{C,i}\;\in R^{n_{fi}}$, and $u_{w,i}\;=\;W_{A,i}\;\in R^{n_{wi}}$ be the states, torques, contact forces, and adjacency wrench, respectively; then, the non-linear dynamics of the robot can be written as in Equation (22). The signal sizes for **Agent 1** are $n_{x1}\;=\;6$, $n_{t1}\;=\;0$, $n_{f1}\;=\;0$, $n_{w1}\;=\;6$, while **Agent 2** and **Agent 3** have signal sizes of $n_{xi}\;=\;9$, $n_{ti}\;=\;3$, $n_{fi}\;=\;3$, and $n_{wi}\;=\;6$ for i=2,3.

(28) $$\begin{array}{cc} {\dot{x}}_{i}\;=\; & f_{i}\left(q_{i},u_{\tau ,i},u_{c,i}\right)\\ f_{i}\;=\; & M_{i}{\left(q_{i}\right)}^{-1}\Theta_{i}\\ \Theta_{i}\;=\; & -C_{i}\left(q_{i},{\dot{q}}_{i}\right)-G_{i}\left(q_{i}\right)\\ \; & +S_{i}\tau_{i}+J_{C,i}^{T}F_{C,i}+\sum_{m}J_{A,m}^{T}W_{A,m} \end{array}$$

## 5. Cooperative Graph-Theoretic Online Trajectory Generation for ASLB

**Figure 6:**
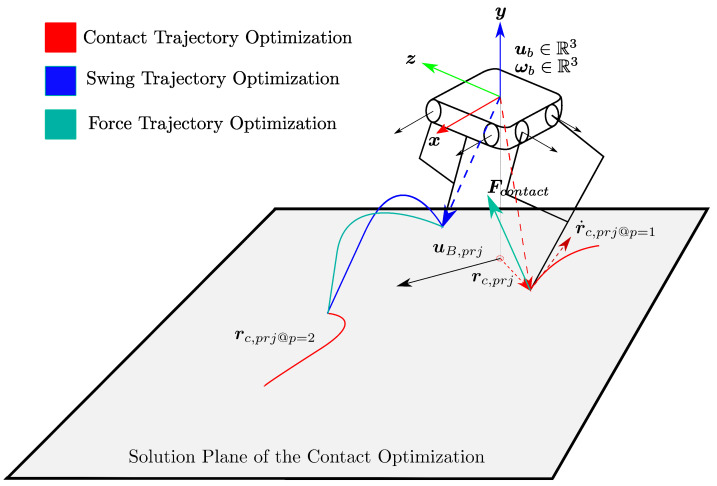
Cooperative interconnection between agents and generalized coordinates at every agent.

Graph-theoretic online trajectory generation relies on the cooperative modeling of the robot and is composed of a series of optimizations. These are the contact phase, swing phase, and force optimizations, where contact optimization finds the optimal finite horizon for the current contact phase and a sequence of contact trajectories that will keep the robot states bounded for defined phase horizon. For this reason, the resultant contact phase trajectories, except for the one associated with the current contact leg, are not passed to the next optimization. Another cardinal data set that is passed to the next optimization from contact phase is the initial point of the subsequent contact phase. This information is required to generate a rough swing trajectory for the leg that is in the air. It should be noted that both contact and swing trajectories are calculated to provide an initial trajectory for the force optimization, where trajectories are refined using a cooperative system framework. Trajectories to previously defined optimizations are illustrated in Figure 6.

### 5.1. Contact Phase Optimization

The contact-phase optimization calculates a set of trajectories using the linear inverted pendulum model (LIPM) and contact constraints. The LIPM dynamics we used in this work are widely used in the vast majority of the literature. As shown in Section 5.3, the dynamics are written with respect to body frame $F_{B}$. Besides that, the dynamics are also kept at the velocity level. Under these circumstances, the contact conditions for the leg in contact need to be defined accordingly.

#### 5.1.1. Contact Condition

Under contact conditions with the no-slip assumption, $r_{c,i}$ has no relative motion with respect to the ground if this condition is observed from the inertial frame $F_{I}$. This condition is observed from $F_{B}$ as if ${\dot{r}}_{c,i}\;=\;-u_{B}$, which is illustrated in Equation (29) for agents i=2,3. Recall that states of agents i=2,3 are denoted as $x_{i}\;=\;{\left[q_{t,i}^{T}\;q_{r,i}^{T}\;q_{a,i}^{T}\right]}^{T},\;\;i=2,3$, and ${\dot{q}}_{t,i}$, where  i=2,3 is duplicate of $u_{B}$, assuming that the connections between nodes are as defined in Equation (26).

(29) $$\begin{array}{cc} 0\; & =\;\left[\begin{array}{ccc} -I & -J_{r,i} & J_{a,i} \end{array}\right]\left[\begin{array}{c} {\dot{q}}_{t,i}\\ {\dot{q}}_{r,i}\\ {\dot{q}}_{a,i} \end{array}\right]\;=\;\left[\begin{array}{ccc} -I & -J_{r,i} & J_{a,i} \end{array}\right]\left[\begin{array}{c} u_{B}\\ {\dot{q}}_{r,i}\\ {\dot{q}}_{a,i} \end{array}\right] \end{array}$$

Assuming the body is slowly rotating, ${\dot{q}}_{r,i}\approx 0,\;\;i=2,3$

#### 5.1.2. LIPM Model

**Figure 7:**
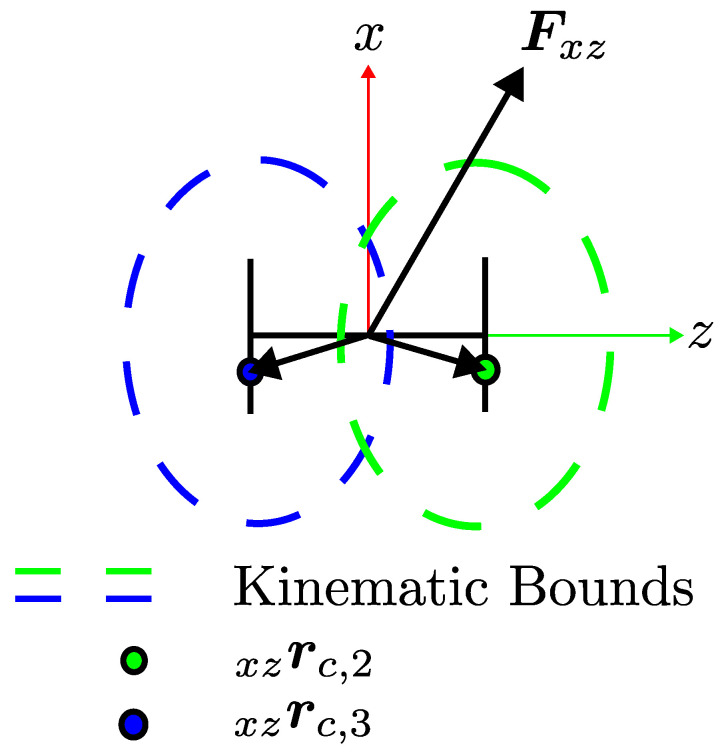
LIPM dynamics projected on the x−z plane.

The implementation of the LIPM model in this work has some nuances compared to the the work where it is proposed [43,44] and illustrated in Figure 7. Let $F_{xz}\in R^{2}$ be the virtual force created on the x−z plane due to the displacement between origin and contact locations rc,2 xz  and rc,3 xz . Note that the origin represents the center of mass (CoM) and is not the origin of the $F_{B}$. Although CoM moves with respect to the origin of $F_{B}$, in practice, it is assumed to be fixed with an offset from $F_{B}$.

Under these assumptions, the LIPM dynamics are provided as in Equation (30) in state space form. States for this system are denoted as $x_{c}$ and defined as the contact point, and this point is denoted as $\Delta r_{xz}$, as any of the two contact locations can be assigned to it, which are rc,2 xz  and rc,3 xz , respectively. Specifically, states are defined as ${\bar{x}}_{c,1}\;=\;\Delta r_{xz}\in R^{2}$, ${\bar{x}}_{c,2}\;=\;\frac{d}{dt}{\Delta r}_{xz}\in R^{2}$, which are combined as ${\bar{x}}_{c}\;=\;{\left[{\bar{x}}_{c,1}^{T}\;\;{\bar{x}}_{c,2}^{T}\right]}^{T}$.

(30) $${\dot{\bar{x}}}_{c}\;=\;\left[\begin{array}{c} {\dot{\bar{x}}}_{c,1}\\ {\dot{\bar{x}}}_{c,2} \end{array}\right]\;=\;\left[\begin{array}{cc} 0 & I\\ \omega_{0}^{2}I & 0 \end{array}\right]\left[\begin{array}{c} {\bar{x}}_{c,1}\\ {\bar{x}}_{c,2} \end{array}\right]\;=\;A_{lipm}{\bar{x}}_{c}$$

This system is an inherently unstable system; therefore, what is being pursued with this system is to find a set of initial conditions, denoted as x¯c  0, that will propel the CoM of the robot toward the desired velocity vector. While carrying that out, a set of state bounds are also satisfied. Equation (30) is discretized using Euler propagation as provided below, where Δt is the sampling time.

(31) $${\bar{x}}_{c,k+1}\;=\;\left(I+\Delta tA_{lipm}\right){\bar{x}}_{c,k}$$

#### 5.1.3. Contact Phase Optimization

This method relies on finding a set of trajectories that will keep the proceeding steps within bounds; therefore, a phase horizon is defined as $N_{p}\in N$, which represents the number of phases to be calculated during the optimization, including the current phase. A finite horizon for each phase is defined as $N_{n}\in N$, which will be minimized for $N_{p}\;=\;1$ and kept at its nominal for $N_{p}\;>\;1$. The states of the LIPM dynamics in each phase are denoted as x¯c,k  p, where $p\;=\;\left[1,\;\cdots ,\;N_{p}\right]$ and $k\;=\;\left[1,\;\cdots ,\;N_{n}\right]$. The combined states for each phase are denoted as xc,K  p as provided below.

(32) x¯c,K  p=x¯c,1  p⋮x¯c,Nn  p,∀p

Equation (33) represents the quadratic problem that runs in contact optimization phase, where Lc(x¯c,1  p,uB,ref) is the objective function, and Ccont(x¯c,1  p) and Cbounds(x¯c,1  p) are equality and inequality constraints to ensure continuity of the states between phases and to keep the states within predefined bounds.

(33) minimizex¯c,1  p∑k=1MLc(x¯c,1  p,uB,ref)s.t.Ccont(x¯c,1  p)=0Cbounds(x¯c,1  p)≤0

#### 5.1.4. Contact Phase: Continuity Constraint

As explained earlier, contact optimization seeks to find several contact phase trajectories, and these trajectories should ideally be continuous. This is achieved by an equality constraint defined as in Equation (34) for $p\;=\;1,\;\cdots ,\;N_{p}-1$.

(34) x¯c,Nn  p=x¯c,1  p+10Ix¯c,Nn  p=0Ix¯c,1  p+1

#### 5.1.5. Contact Phase: Constraint for State Bounds

State bounds are defined based on the leg in contact; therefore, state bounds are switching between the bounds of **Agent 2** and **Agent 3**. Let $c_{2}\;=\;\left[0,1\right]$ and $c_{3}\;=\;\left[0,1\right]$ be the contact indicators of **Agent 2** and **Agent 3**, respectively. Under contact $c_{i}\;=\;1$; otherwise, $c_{i}\;=\;0$ for i=2,3 at phase *p*. The bounds for **Agent 2** and **Agent 3** are defined as $S_{i}\;=\;{\left[S_{i,UB}^{T},\;S_{i,LB}^{T}\right]}^{T}$ and assigned to $S_{p}$ such that $S_{p}\;=\;S_{i}$ if $c_{i}=1$. Note that we are assuming a single point of contact. Using the bounds, the state boundary constraints are defined as in Equation (35).

(35) I−II0x¯c,k  p≤Sp,UBSp,LB

#### 5.1.6. Contact Phase: Cost Function

Contact phase optimization aims to reach the x−z projection of reference velocity that is provided by the user $u_{B,ref}$, which is $u_{B,xz}$. Recall that ${\dot{r}}_{c,i}\;=\;-u_{B}$; therefore, the cost function is written as in Equation (36).

(36) Jcontact=uB,xz+0Ix¯c,K  pTQsuB,xz+0Ix¯c,K  pT+0Ix¯c,K  pTQp0Ix¯c,K  pT

### 5.2. Swing Phase Optimization

**Figure 8:**
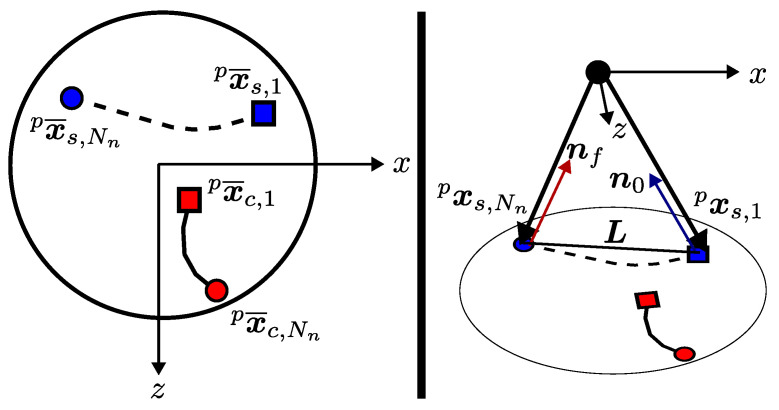
Swing phase trajectory generation.

Swing phase optimization calculates a rough trajectory for the swinging leg by connecting the current position of the tip of the swinging leg to the initial point of the proceeding contact phase trajectory with a bezier curve. Instances of the swing trajectory are denoted as xs,k  p, and the current and final positions of the swing trajectory are denoted as xs,1  p and xs,Nn  p, respectively. x¯s,1  p and x¯s,Nn  p are the projections of the vectors on the x−z plane, and the initial point of the proceeding contact phase trajectory is x¯c,1  p+1. Note that the contact phase optimization generates a planar trajectory. Therefore, the implementation of swing-phase trajectory optimization requires a modification of these vectors by adding the height of the CoM to the *y* axis of any projected vector if it needs to be passed to the swing trajectory. Figure 8 illustrates previously mentioned vectors. Dashed lines represent the projection of the swing trajectory, while solid lines show the contact trajectory. $n_{0}$, $n_{f}$, and L represent the unit vector to CoM, the unit vector from CoM, and the straight line on the x−z plane between initial and final positions of the swing trajectory. Formal definitions for $n_{0}$, $n_{f}$ and L are provided in Equation (37).

(37) n0=−xs,1  p=1|xs,1  p=1|nf=xs,Nn  p=1|xs,Nn  p=1|L=xs,Nn  p=1−xs,1  p=1

#### 5.2.1. Swing Phase Optimization

Swing phase optimization is run for the current phase; therefore, unlike for the contact phase optimization, p=1. The finite horizon for this phase is the $N_{n}$ at p=1. Note that at p=1, $N_{n}$ is optimized at the contact phase optimization.

Let $b_{x,j}$, $b_{y,j}$, and $b_{z,j}$ be the coefficients of the bezier curve, where j=1,⋯,nb. $c_{b}$ and $v_{b}$, which are sorted collections of $b_{x,j}$, $b_{y,j}$, and $b_{z,j}$, are defined. This classification collects coefficients related to initial and final positions of the curve under $c_{b}$ and coefficients that are being optimized under $v_{b}$. Specifically $c_{b}$ is defined as cb=xs,1T p=1xs,Nn  p=1T. Based on thepreviously described notation, bezier curves for swing trajectory are defined as in Equation (39), where $J_{b,c}$ and $J_{b,v}$ are matrix-valued functions of *k*, which can be populated for $k=1<\cdots ,N_{n}$ and maps $c_{b}$ and $v_{b}$ to xs,k  p=1∈R3. Similarly, $c_{b}$ and $v_{b}$ are mapped to x˙s,k  p=1∈R3 using Jb,c d  and Jb,v d .

Equation (38) represents the quadratic problem that runs in the swing optimization phase, where $L_{s}\left(v_{b}\right)$ is the objective function and $S_{bounds}\left(v_{b}\right)$ is the set of inequality constraints to keep states within predefined bounds.

(38) $$\begin{array}{ccccc} \; & \underset{v_{b}}{\mathrm{minimize}} & \; & \sum_{k=1}^{M}L_{s}\left(v_{b}\right) & \\ \; & s.\;t. & \; & C_{bounds}\left(v_{b}\right)\;\leq \;0 \end{array}$$

(39) xs,k  p=1=Jb,c(k)Jb,v(k)cbvbx˙s,k  p=1=Jb,c d (k)Jb,v d (k)cbvb

The implementation of swing phase optimization requires the calculation of Jb,c d0, Jb,c df, Jb,v d0, Jb,v df using Equation (39) at k=1 and $k=N_{n}$.

#### 5.2.2. Swing Phase: Constraint for State Bounds

State bounds are defined based on the leg in the swing; therefore, state bounds switch between the bounds of **Agent 2** and **Agent 3**. Bounds for **Agent 2** and **Agent 3** are defined as $S_{i}\;=\;{\left[S_{i,UB}^{T},\;S_{i,LB}^{T}\right]}^{T}$ and assigned to $S_{p}$ such that $S_{p}\;=\;S_{i}$ if $c_{i}=0$. Using these bounds, state boundary constraints are defined as in Equation (40).

(40) $$\begin{array}{cc} \left[\begin{array}{c} \;J_{b,c}\left(k\right)c_{b}+J_{b,v}\left(k\right)v_{b}\\ -J_{b,c}\left(k\right)c_{b}-J_{b,v}\left(k\right)v_{b} \end{array}\right]\; & \leq \;S_{p}\\ \left[\begin{array}{c} J_{b,v}\left(k\right)\\ -J_{b,v}\left(k\right) \end{array}\right]v_{b}\; & \leq \;S_{p}-\left[\begin{array}{c} \;J_{b,c}\left(k\right)\\ -J_{b,c}\left(k\right) \end{array}\right]c_{b} \end{array}$$

#### 5.2.3. Swing Phase: Cost Function

Swing phase optimization aims to pull the swinging leg to the CoM in the beginning of the swing motion and then pushes it toward the final position. Along with these, it also tries to approach the straight line L. Therefore, the cost function of the swing phase optimization is written as in Equation (41).

(41) Jswing=n0−Jb,c d0Jb,v d0cbvbTQ0n0−Jb,c d0Jb,v d0cbvb+nf−Jb,c dfJb,v dfcbvbTQfnf−Jb,c dfJb,v dfcbvb+L−Jb,cJb,vcbvbTQtL−Jb,cJb,vcbvb

### 5.3. Cooperative Force Optimization

Approximate trajectories are obtained in contact and swing phase optimizations, and these trajectories are used in the cooperative force optimization as the initial trajectory. In order to follow the method easily, agent dynamics are rewritten in Equation (43), where $M_{h}$ and $C_{h}$ are partitioned as given in Equation (42). In addition, $M_{i}$, $C_{i}$, and $G_{i}$ for i=2,3 are assigned to $M_{h}$, $C_{h}$ and $G_{h}$, where h=S represents swing, h=C represents contact, and h=B represents floating base matrices. Minimal representation of the dynamics are provided in Equation (44). Floating base dynamics do not switch; however, **Agent 2** and **Agent 3** dynamics are assigned to h=C or h=S depending on $c_{i}$ for i=2,3. Similarly, wrenches $W_{A,m}$ are assigned to $W_{A,h}$ based on $c_{i}$ such that if $c_{2}=1$, then $W_{A,C}\;=\;W_{A,2}$ and if $c_{3}=1$, then $W_{A,C}\;=\;W_{A,3}$ for i=2,3. Finally, ${\tilde{r}}_{C}$ is the tip point position $r_{c,i}$ of the leg with $c_{i}=1$. $F_{C}$ is the interaction between the contact leg and the ground.

(42) Mh=Mh bb Mh bq Mh qb Mh qq Ch=Ch bb Ch bq Ch qb Ch qq 

(43) MB bb x˙B+CB bb xB+GB bb =W¯A,C+W¯A,SMS bb MS bq x˙S+CS bb CS bq xS+GS bb =WA,SMC bb MC bq x˙C+CC bb CC bq xS+GC bb =WA,C+Ir˜CFC

(44) $$\begin{array}{cc} {\hat{M}}_{B}{\dot{x}}_{B}+{\hat{C}}_{B}x_{B}+{\hat{G}}_{B}\; & =\;{\bar{W}}_{A,C}+{\bar{W}}_{A,S}\\ {\hat{M}}_{S}{\dot{x}}_{S}+{\hat{C}}_{S}x_{S}+{\hat{G}}_{S}\; & =\;W_{A,S}\\ {\hat{M}}_{C}{\dot{x}}_{C}+{\hat{C}}_{C}x_{S}+{\hat{G}}_{C}\; & =\;W_{A,C}+\left[\begin{array}{c} I\\ {\tilde{r}}_{C} \end{array}\right]F_{C} \end{array}$$

Then, continuous models in Equation (43) are converted into discrete models using Euler discretization, and Equation (45) provides the discrete system model that is used in cooperative force optimization. The current states are denoted as $x_{h,k}$, where *h* and *k* represent the model identifier and the prediction step, respectively.

(45) $$\begin{array}{c} \left[\begin{array}{cc} \Delta t{\hat{C}}_{B,k}-{\hat{M}}_{B,k} & {\hat{M}}_{B,k} \end{array}\right]\left[\begin{array}{c} x_{B,k}\\ x_{B,k+1} \end{array}\right]-\Delta t{\bar{W}}_{S,k}-\Delta t{\bar{W}}_{C,k}\;=\;\Delta t{\hat{G}}_{B}\\ \left[\begin{array}{cc} \Delta t{\hat{C}}_{S,k}-{\hat{M}}_{S,k} & {\hat{M}}_{S,k} \end{array}\right]\left[\begin{array}{c} x_{S,k}\\ x_{S,k+1} \end{array}\right]-\Delta tW_{S,k}\;=\;\Delta t{\hat{G}}_{S,k}\\ \left[\begin{array}{cc} \Delta t{\hat{C}}_{C,k}-{\hat{M}}_{C,k} & {\hat{M}}_{C,k} \end{array}\right]\left[\begin{array}{c} x_{C,k}\\ x_{C,k+1} \end{array}\right]-\Delta tW_{C,k}-\Delta t{\hat{J}}_{C,k}F_{C,k}\;=\;\Delta t{\hat{G}}_{C,k} \end{array}$$

For brevity, Equations (45) are represented with a minimal representation as follows.

(46) $$\begin{array}{cc} {\tilde{M}}_{h,k}\; & =\;\left[\begin{array}{cc} {\tilde{M}}_{h_{1},k} & {\tilde{M}}_{h_{2},k} \end{array}\right]\;=\;\left[\begin{array}{cc} \Delta t{\hat{C}}_{B,k}-{\hat{M}}_{B,k} & {\hat{M}}_{B,k} \end{array}\right]\\ {\tilde{J}}_{C,k}\; & =\;-\Delta t{\hat{J}}_{C,k}\\ {\tilde{G}}_{h,k}\; & =\;\;\Delta t{\hat{G}}_{h,k}\\ {\tilde{P}}_{h,k}\; & =\;-\Delta t\hat{I} \end{array}$$

#### 5.3.1. Cooperative Force Optimization Problem

This method relies on initially provided trajectories that is provided by contact and swing phase. In this phase, decision variables are defined as corrections to the nominal trajectories, and a complete trajectory is defined as such. Nominal trajectories for states are denoted with xh,k 0 , and corrections to the nominal trajectories at every instant are denoted as $\Delta x_{h,k}$. Similarly, force trajectories are defined in the same fashion such that FC,k 0  and WA,m 0  are the nominal force trajectories, while $\Delta F_{C,k}$ and $\Delta W_{A,m}$ are the corrections to the relevant trajectories.

(47) xh,kT=xh,k 0 +Δxh,kFC,kT=FC,k 0 +ΔFC,kWh,kT=Wh,k 0 +ΔWh,k

States, contact forces, and wrenches for the entire trajectory are combined as follows.

(48) $$\begin{array}{cc} x_{h,K}^{T}\; & =\;{\left[\begin{array}{ccc} x_{h,1}^{T} & \cdots & x_{h,N_{n}}^{T} \end{array}\right]}^{T}\\ F_{C,K}^{T}\; & =\;{\left[\begin{array}{ccc} F_{C,1}^{T} & \cdots & F_{C,N_{n}}^{T} \end{array}\right]}^{T}\\ W_{h,K}^{T}\; & =\;{\left[\begin{array}{ccc} W_{h,1}^{T} & \cdots & W_{h,N_{n}}^{T} \end{array}\right]}^{T} \end{array}$$

The relationship between $x_{h,K}$, $F_{C,K}$, and $W_{h,K}$ can be written for the entire trajectory using combined matrices as provided in Equation (49).

(49) $$.\begin{array}{cc} {\tilde{M}}_{h,K}\; & =\;\left[\begin{array}{cccc} {\tilde{M}}_{h_{1},1} & {\tilde{M}}_{h_{2},2} & 0 & \cdots \\ 0 & {\tilde{M}}_{h_{1},2} & {\tilde{M}}_{h_{2},3} & \\ \vdots & \; & \ddots & \ddots \end{array}\right]\\ {\tilde{J}}_{C,K}\; & =\;\left[\begin{array}{ccc} -\Delta t{\hat{J}}_{C,1} & 0\; & \cdots \\ 0 & -\Delta t{\hat{J}}_{C,2} & \\ \vdots & \; & \ddots \end{array}\right]\\ {\tilde{G}}_{h,K}\; & =\;\left[\begin{array}{c} {\tilde{G}}_{h,1}\\ {\tilde{G}}_{h,2}\\ \vdots \end{array}\right]\\ {\tilde{P}}_{h,K}\; & =\;\left[\begin{array}{ccc} {\tilde{P}}_{h,1} & 0 & \cdots \\ 0 & {\tilde{P}}_{h,2} & \\ \vdots & \; & \ddots \end{array}\right] \end{array}$$

The dynamics for the floating base, contacting, and swinging bodies are written as provided in Equation (50).

(50) $$\begin{array}{c} \left[\begin{array}{ccc} {\tilde{M}}_{B,K} & {\tilde{P}}_{S,K} & {\tilde{P}}_{C,K} \end{array}\right]\left[\begin{array}{c} x_{B,K}\\ {\bar{W}}_{S,K}\\ {\bar{W}}_{C,K} \end{array}\right]\;=\;G_{B,K}\\ \left[\begin{array}{cc} {\tilde{M}}_{S,K} & {\tilde{P}}_{S,K} \end{array}\right]\left[\begin{array}{c} x_{S,K}\\ W_{S,K} \end{array}\right]\;=\;G_{S,K}\\ \left[\begin{array}{ccc} {\tilde{M}}_{C,K} & {\tilde{P}}_{C,K} & {\tilde{J}}_{C,K} \end{array}\right]\left[\begin{array}{c} x_{C,K}\\ W_{C,K}\\ F_{C,K} \end{array}\right]\;=\;G_{C,K} \end{array}$$

The optimization for the three agents is represented in a single objective Equation (51) and a set of constraints in this work; however, the problem is readily available for distributed optimization.

(51) $$\begin{array}{ccccc} \; & \underset{\Delta x_{h,k},\Delta F_{C,k},\Delta W_{h,k}}{\mathrm{minimize}} & \; & \sum_{k=1}^{M}L_{c}\left(x_{h,k},F_{C,k},W_{h,k}\right) & \\ \; & s.\;t. & \; & C_{dyn}\left(\Delta x_{h,k},\Delta F_{C,k},\Delta W_{h,k}\right)\; & =\;0\\ \; & \; & & C_{coop}\left(\Delta x_{h,k},\Delta F_{C,k},\Delta W_{h,k}\right)\; & =\;0\\ \; & \; & & C_{cntct}\left(\Delta x_{h,k}\right)\; & =\;0\\ \; & \; & & C_{fc}\left(\Delta F_{C,k}\right)\; & \leq \;0 \end{array}$$

Based on the dynamics given in Equation (49) and the representation of the trajectories provided in Equation (47), the matrices for the equality constraints are denoted as $A_{h,dyn}$ and $B_{h,dyn}$. The equality constraint is provided for only the floating base for brevity.   

(52) M˜B,KP˜S,KP˜C,KΔxB,KΔW¯S,KΔW¯C,K=GB,K−M˜B,KP˜S,KP˜C,KxB,K 0 W¯S,K 0 W¯C,K 0 

#### 5.3.2. Cooperative Force Optimization: Contact Constraint

The contact constraint was provided previously in Equation (29). This condition is modified for the definition of the trajectory provided in Equation (47). Put simply, the contact point velocity has to be equal to the body velocity in the opposite direction in the no-slip condition, and Equation (53) projects the relationship on the decision variables for the quadratic optimization.

(53) 0=−IJa,ixB,K0=−IJa,iΔxB,K+−IJa,ixB,K 0 −−IJa,iΔxB,K=−IJa,ixB,K 0 

#### 5.3.3. Cooperative Force Optimization: Force Cone Constraint

**Figure 9:**
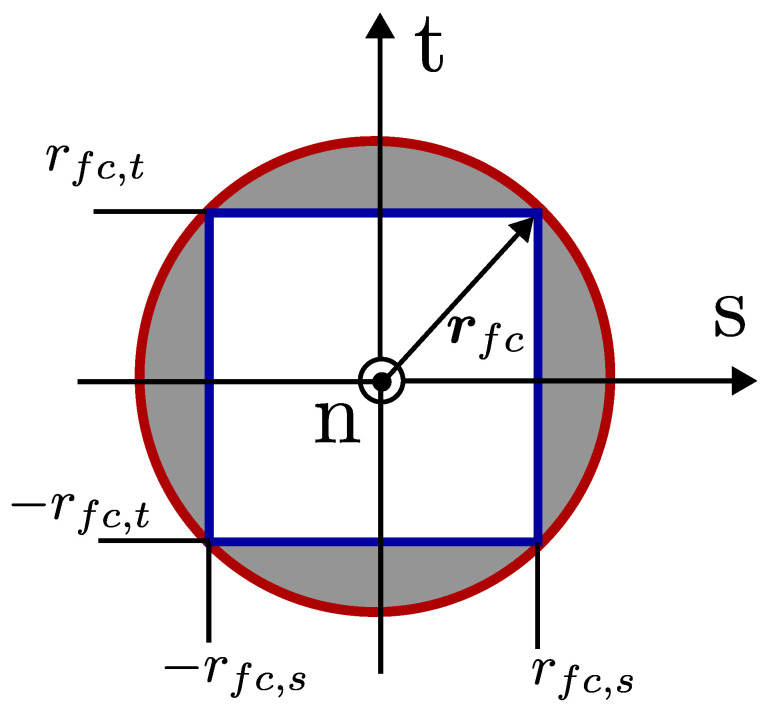
Geometric interpretation of the friction pyramid.

The coooperative force optimization phase is designed as a quadratic problem; therefore, constraints have to be set accordingly. Contact constraints are dedicated to keep tangential forces small so that no slipping occurs. To do so, a friction pyramid is created inside a friction cone. The friction cone is a geometric interpretation of the magnitude of the allowable tangential force that can be applied on the ground. The allowable limit is calculated by simply multiplying the normal component of the applied force by the contact point with the friction coefficient. The nrmal component of the force is denoted as fc,n B  and the tangential components of the applied force are denoted as fc,t B , fc,s B , respectively. It should be noted that the applied force is defined with respect to $F_{B}$. The friction coefficient is denoted as μ. The radius of the friction cone is defined as rfc=μfc,n B . The friction pyramid is defined such that it is always upper-bounded by the $r_{fc}$, and this is achieved by setting linear bounds that are denoted as $r_{fc,s}$ and $r_{fc,t}$. These bounds are calculated such that $|r_{fc,s}|\;\approx \;0.707r_{fc}$ and $|r_{fc,s}|\;\approx \;0.707r_{fc}$. It should be noted that the bounding $r_{fc}$ creates a non-linear relationship and makes optimization a non-linear problem; however, bounds that are defined for tangential components can be implemented in a linear fashion. The geometric interpretation of the friction cone (red solid line) and pyramid (blue solid line) is provided in Figure 9.

Based on the linear and conservative bounds, the following linear constraints are defined for the tangential forces.

(54) −0.707μfc,n B ≤fc,t B ≤0.707μfc,n B −0.707μfc,n B ≤fc,s B ≤0.707μfc,n B −2×0.707μfc,n B ≤fc,t B +fc,s B ≤2×0.707μfc,n B 

The relationship given in Equation (54) is written compactly as provided in Equation (55), where FC,k B  is the vector containing the decision variables. n  B , t  B  and s  B  are the unit vectors attached on the contact points and defined in $F_{B}$. As a practical note, calculating these unit vectors with respect to $F_{B}$ is a straightforward calculation when there are passive joints at the ankle of the contact legs. Depending on the kinematic structure of the leg, certain unit vectors can be assumed to be in the same direction with the axes of the body frame.

(55) −2×0.707μn TB −t TB −s TB FC,k B ≤02×0.707μn TB +t TB +s TB FC,k B ≤0

Within the current work, n  B , t  B  and s  B  are assumed to be constant and defined as n TB =010T, t  B =100T and s  B =001T.

#### 5.3.4. Cooperative Force Optimization: Cost Function

Force phase optimization tries to keep the commanded motion intact while minimizing the disturbance injected on the system due to the joint accelerations. The joint accelerations affect each agent due to the $C_{coop}$, which is provided in Equation (26). The constraints that are introduced in this chapter previously maintained contact, and force cone constraints are satisfied. The cost function for this optimization is defined in Equation (56).

(56) $$\begin{array}{cc} \; & L_{c}\left(x_{h,k},F_{C,k},W_{h,k}\right)\;=\;\\ & {\left(-u_{B,xz}+\left[\begin{array}{ccccc} 1 & 0 & 0 & 0 & 0\\ 0 & 0 & 1 & 0 & 0 \end{array}\right]x_{B,k}\right)}^{T}Q_{T}\left(-u_{B,xz}+\left[\begin{array}{ccccc} 1 & 0 & 0 & 0 & 0\\ 0 & 0 & 1 & 0 & 0 \end{array}\right]x_{B,k}\right)+\\ & x_{B,k}^{T}{II}_{Q}^{T}Q_{W}{II}_{Q}x_{B,k} \end{array}$$

where ${II}_{Q}$ is a matrix that selects the states that are the joint velocities of contact and swing legs and approximate the acceleration of these selected states. The joint velocities within states $x_{h,k}$ are denoted as $q_{h,k}$ for h=C,S, and the selection of these states is defined in Equation (57).

(57) $$\begin{array}{cc} {\dot{q}}_{h,k}\;= & \;\left[\begin{array}{ccccc} 0 & 0 & 1 & 0 & 0\\ 0 & 0 & 0 & 1 & 0\\ 0 & 0 & 0 & 0 & 1 \end{array}\right]x_{h,k}\\ {\dot{q}}_{h,k}\;= & \;{II}_{q}x_{h,k} \end{array}$$

The approximation for acceleration is given below, where states are assumed to propagate with the first-order Euler method.

(58) $${\ddot{q}}_{h,k}\;=\;{II}_{q}\frac{\left(x_{h,k+1}-x_{h,k}\right)}{\Delta t}$$

Finally, accelerations ${\ddot{q}}_{h,k}$ for h=C,S for $k=1,\cdots ,N_{n}$ is given as provided in Equation (59)   

(59) $$\begin{array}{cc} {\ddot{q}}_{h,k}\;= & \;\left[\begin{array}{cccc} -\Delta t{II}_{q} & \Delta t{II}_{q} & \; & \\ \; & -\Delta t{II}_{q} & \Delta t{II}_{q} & \\ \; & \; & & \ddots \end{array}\right]\left[\begin{array}{c} x_{h,1}\\ x_{h,2}\\ \vdots \\ x_{h,N_{n}} \end{array}\right]\\ {\ddot{q}}_{h,k}\;= & \;{II}_{Q}x_{h,K} \end{array}$$

## 6. Preliminary Results for the ASLB Platform

This section presents the simulation of the algorithm for the proposed method. The simulation is not executed in a physics environment, and trajectories illustrated in this section are estimated trajectories only. Solutions are obtained on Intel(R) Core i7-4720HQ CPU @2.60 GHz 16GB RAM PC with MATLAB 2019b software.

The sampling time Δt for the discrete model is selected to be Δt=0.05s. Nominal values for $N_{n}$ and $N_{p}$ are selected as $N_{n}\;=\;20$ and $N_{p}\;=\;4$, respectively. $Q_{s}$ and $Q_{p}$ for contact phase optimization is selected to be $Q_{s}\;=\;5$ and $Q_{p}\;=\;2$. $Q_{0}$, $Q_{f}$, and $Q_{t}$ for swing phase optimization is selected to be $Q_{0}\;=\;12.5$, $Q_{f}\;=\;12.5$, and $Q_{t}\;=\;30$. Finally, $Q_{u}$ and $Q_{w}$ for cooperative force optimization are selected to be $Q_{u}\;=\;100$ and $Q_{w}\;=\;5$.

Based on these settings, and from the initial conditions of $u_{B}\left(t=0\right)\;=\;{\left[\begin{array}{ccc} 0 & 0 & 0 \end{array}\right]}^{T}$, $r_{2}\left(t=0\right)\;=\;{\left[\begin{array}{ccc} 0.01 & -0.344 & 0.063 \end{array}\right]}^{T}$, and $r_{3}\left(t=0\right)\;=\;{\left[\begin{array}{ccc} 0.045 & -0.344 & -0.239 \end{array}\right]}^{T}$, ASLB is asked to move forward by $u_{B,ref}\;=\;{\left[\begin{array}{ccc} 0.1 & 0 & 0 \end{array}\right]}^{T}$. The results are provided in the following figures.

**Figure 10:**
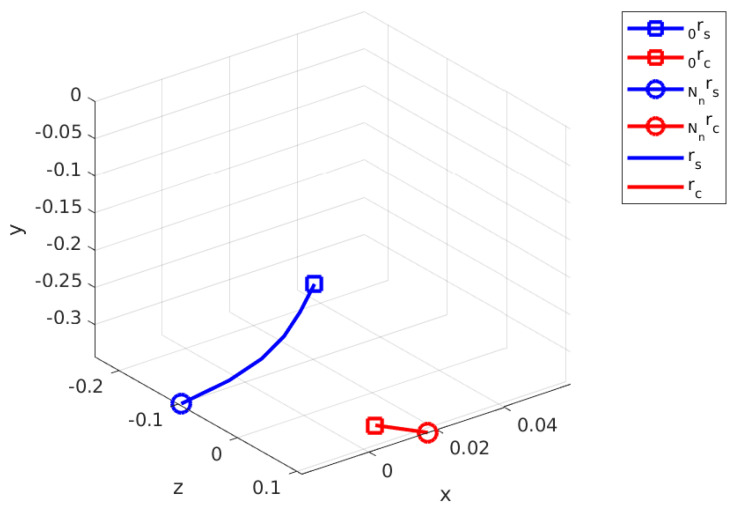
Calculated swing and contact leg trajectories for first step.

Figure 10 illustrates the calculated swing and contact trajectories. $N_{n}$ for the first optimization is minimized to $N_{n}\;=\;6$ from a nominal 20 as the contact initial position for rc,1  p=1 is already provided by the sensor information. For a feasible finite horizon, $N_{n}=6$, and contact leg, which is the right leg or **Agent 2**, is used to calculate an approximate trajectory for $r_{c}$ starting from $r_{2}\left(t=0\right)$ and diverge from the CoM toward the (+) x and (+) z directions. Note that this trajectory is calculated with respect to $F_{B}$. The swing phase optimization connects $r_{3}\left(t=0\right)$ to the rc,1  p=2 without violating the kinematic bounds.

**Figure 11:**
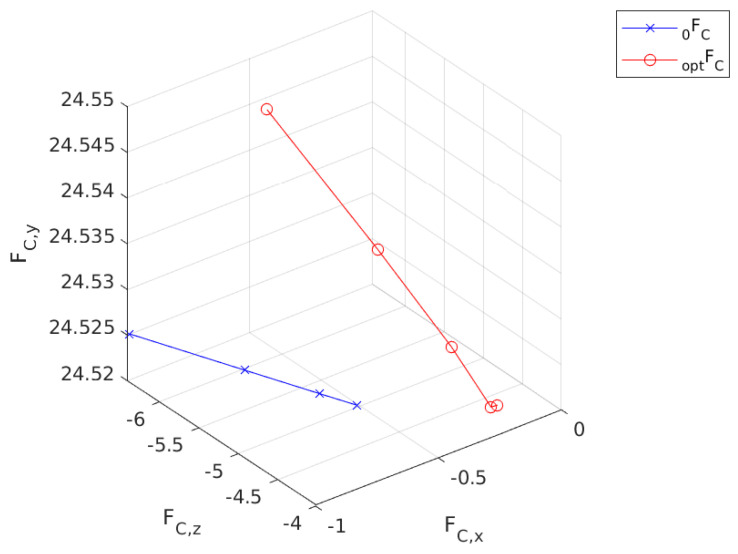
Calculated force trajectory for the first step.

Figure 11 illustrates the nominal force trajectory for the contact leg as it interacts with the ground. FC 0  is the initial trajectory that is calculated by substituting $q_{a,i}$ and $x_{i}$ into the agent dynamics. FC opt  is the resultant contact force trajectory. It is visible from the figure that there is not a significant change in the y-axis. However, FC opt  is shifted in the (+) x direction by approximately 0.4 N.

**Figure 12:**
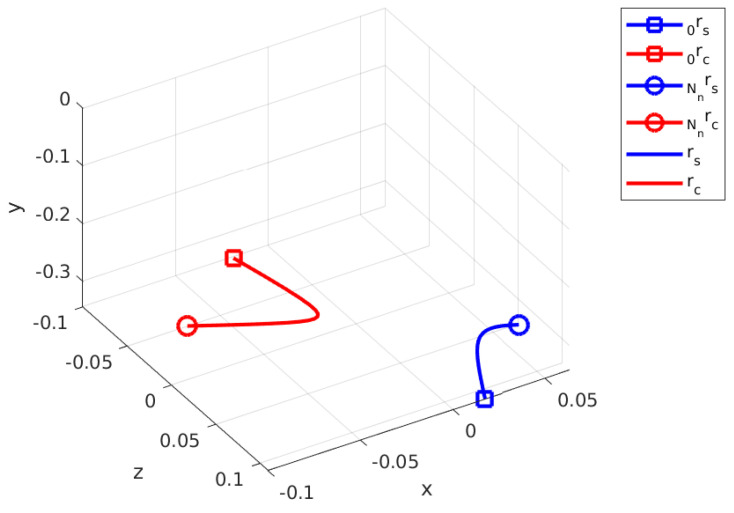
Calculated swing and contact leg trajectories for the second step.

Figure 12 shows the contact and swing trajectories for the second step, where $N_{n}$ is minimized to $N_{n}\;=\;16$. Similar to the first step, rc,1  p=1 for contact phase optimization is the initial position of the current contact leg, which is **Agent 3**. $r_{c}$ in Figure 12 converges to the CoM in the beginning of this phase and then pushes away toward the (-) x and (-) z directions.

**Figure 13:**
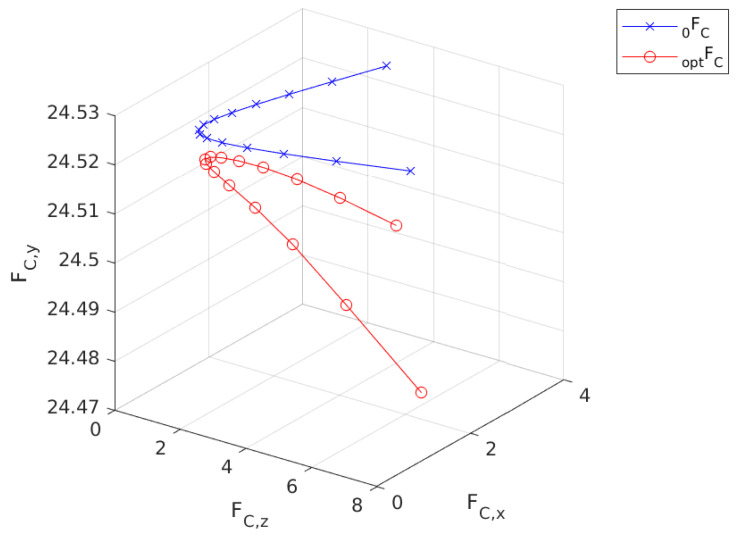
Calculated force trajectory for the second step.

A smaller correction occurs in the contact force as FC 0  and FC opt  have slight differences in all three directions as seen in Figure 13.

**Figure 14:**
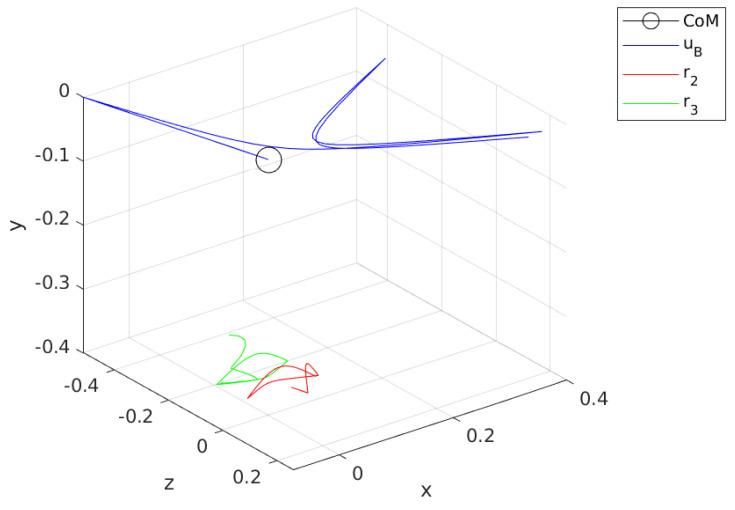
$u_{B}$ trajectory plot along with leg motion with respect to CoM.

Figure 14 provides the $u_{B}$ trajectory throughout the walking simulation along with the $r_{c,i}$ for i=2,3. This figure illustrates the characteristic difference in the method, which calculates the contact positions and forces them to track a reference velocity $u_{B,ref}$. The black circle in Figure 14 indicates the CoM and decision variables are defined with respect to that. The trajectory of $u_{B}$, which is given with the blue line, settles in a cyclic pattern. The mean of the magnitude in x direction is approximately 0.28 m/s, while it is almost zero for z direction.

**Figure 15:**
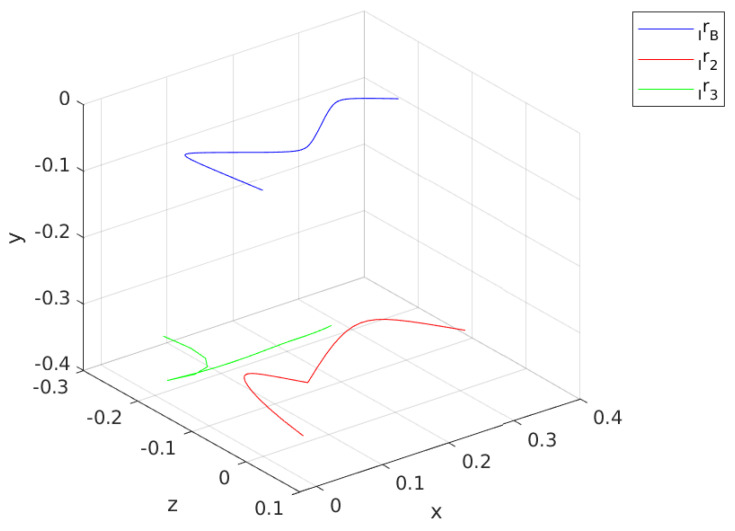
$r_{B}$ plot along with leg motion with respect to $F_{I}$.

Figure 15 provides a more intuitive illustration of the motion of the robot as rB I  is calculated from $u_{B}$. rB I  gives the position of the origin of the $F_{B}$ with respect to the $F_{I}$. The trajectory of rB I  reveals that body drifts away from the line z=0 while achieving forward motion, as desired. The drift is due to the first contact leg, which is the **Agent 2**, and cannot be corrected.

With the proposed method, a velocity command could be tracked with some offset. During the simulation of the algorithm, it was observed that relaxation of the state bounds decreases the offsets between velocity commands and actual velocity. In addition, since this method is designed for velocity level dynamics, a drift in the walking does occur, which can be mitigated by an appropriate control scheme. That said, extracting the dynamics from the kinematics and developing a controller for this simpler set equations allows faster calculation of the future steps.

## 7. Cooperative UAV-UGV Docking with Task Prioritization

Motivated by the results from the preceding section, we extend the framework to study systems that are not on the same platform for example a cooperative UAV-UGV system similar to that in [39], with the objective to have the UAV (quadcopter) dock on the moving UGV (rover). Note that this framework further illustrates that it can accommodate very different dynamical systems, unlike the ASLB system, where the left and right leg dynamics were identical.

Table 2 provides a summary of the key variables and terms used for the development of the ASLB dynamical model.

### 7.1. Quadcopter Dynamics

The six-degree-of-freedom (DoF) rigid body dynamics with mass ($m_{q}$) and moment of inertia ($J_{q}$) of the quadcopter are represented in (60). The model’s states are denoted as $x_{q}={\left[\begin{array}{cccc} p_{q}^{T} & v_{q}^{T} & \theta_{q}^{T} & w_{q}^{T} \end{array}\right]}^{T}\in R^{12}$, where pq=pq I ∈R3, vq=vq I ∈R3, $\theta_{q}\in R^{3}$ and wq=wq B ∈R3 are inertial position, inertial velocity, Euler angles, and angular velocity, respectively. Given the Euler angles, the rotation matrix for the quadcopter is denoted as Cq I (θq)=Cq I . The inputs of the system are denoted as $u_{q}={\left[\begin{array}{cc} f_{q} & t_{q}^{T} \end{array}\right]}^{T}\in R^{4}$, where $f_{q}$ is the total thrust generated by the motors on $F_{B}$ and $t_{q}\in R^{3}$ is the column matrix of moments generated on the body defined in $F_{B}$. $E_{q}\left(\theta_{q}\right)$ is the mapping between $w_{q}$ and ${\dot{\theta }}_{q}$ such that $w_{q}=E_{q}\left(\theta_{q}\right){\dot{\theta }}_{q}$ for pre-defined rotation sequence. The quadcopter properties are taken from the work [45].

(60) x˙q=p˙qv˙qe˙qw˙q=f(xq,uq)=vqgu3−(1/mq)Cq I fqu3E−1(θq)wqJq−1(−wq×Jqwq+tq)

### 7.2. Rover Dynamics

The dynamics of the rover are calculated with mass $m_{r}$ and moment of inertia $J_{r}$, assuming that it runs on a flat surface and provided in (61). Based on this assumption, model states are denoted as $x_{r}\;=\;{\left[p_{r,x}\;p_{r,y}\;\Psi_{r}\;v_{r,x}\;w_{r,z}\right]}^{T}$, where $p_{r,x}$ and $p_{r,y}$ represent inertial position on the x−y plane, $\Psi_{r}$ is the Euler angle about the *z* axis, vr,x B =vr,x is the horizontal velocity of the rover, and wr,z B =wr,z is the angular velocity of the rover. The composite rotation matrix for the rover is denoted as Cr I =Cr I (Ψr). Inputs to the system are denoted as $u_{r}\;=\;{\left[f_{r,1}\;f_{r,2}\right]}^{T}$, which represent the traction forces applied on the surface by a set of wheels on the right- and left-hand sides of the rover, respectively. $m_{r}$ is taken as 1 kg, and $J_{r}=diag\left\{0.1\right\}$ kg·m^2^.

(61) x˙r=p˙r,xp˙r,yΨ˙rv˙r,xw˙r,z=f(xr,ur)=u1TCr I vr,xu1u2TCr I vr,xu1wr,z(1/mr)(fr,1+fr,2)J−1(r1×fr,1+r2×fr,2)

## 8. Cooperative Model-Predictive Control Methodology

This section introduces a unified MPC strategy to maintain a docking approach for the long range and a finer docking maneuver in the short range by accommodating a non-linear and a linear MPC designed with edge weight information and task prioritization. This section is divided into four subsections, where non-linear MPC (NMPC), linear MPC (LMPC), cooperative task prioritization, and implementation of the control strategy are described in Section 8.1, Section 8.2, Section 8.3, and Section 8.4, respectively.

The introduced method can be applied on all of the agents as the formulation only considers local neighbor information; therefore, formulations will be provided for the agent denoted as *i*, which is defined by the states $x_{i}\left(t\right)\in R^{n_{x}}$, inputs $u_{i}\left(t\right)\in R^{n_{u}}$, and outputs $y_{i}\left(t\right)\in R^{n_{y}}$. Given the states and inputs, the non-linear dynamics of agent *i* are given in the following form:

(62) $$\begin{array}{cc} {\dot{x}}_{i}\left(t\right) & \;=\;f_{i}\left(x_{i}\left(t\right),\;u_{i}\left(t\right)\right)\\ y_{i} & \;=\;h_{i}\left(x_{i}\left(t\right),\;u_{i}\left(t\right)\right) \end{array}$$

Equation (63) provides the discrete linear representation of the agents’ dynamics. This is obtained by linearizing the current state and input, which is denoted as $\left(x_{i,c},\;u_{i,c}\right)$ and then by converting the continuous time system in a discrete system using Euler discretization (see [46]).

(63) $$\begin{array}{cc} \Delta x_{i,k+1} & \;=\;A_{i}\Delta x_{i,k}+B_{i}\Delta u_{i,k}\\ \Delta y_{i,k} & \;=\;C_{i}\Delta x_{i,k}+D_{i}\Delta u_{i,k} \end{array}$$

where *k* is the current sample such that $\Delta x_{i,k}\;=\;x_{i,k}-x_{i,c}$, and $\Delta u_{i,k}\;=\;u_{i,k}-u_{i,c}$. In this work, we assumed that full state information is shared among the agents. Therefore, matrices $C_{i}$ and $D_{i}$ are assumed to be I and 0, respectively, with compatible sizes.

### 8.1. Non-Linear MPC Formulation

Equation (64) represents the non-linear problem that runs in NMPC where $L_{i}\left(x_{i,k},\;u_{i,k},\;x_{i,ref}\right)$ is the objective function, and $C_{eq}\left(x_{i,k},\;u_{i,k}\right)$ and $C_{ineq}\left(x_{i,k},\;u_{i,k}\right)$ are equality and inequality constraints, respectively. *M* in (64) denotes the prediction horizon for the NMPC.

(64) $$\begin{array}{ccccc} \; & \underset{x_{i,k},u_{i,k}}{\mathrm{minimize}} & \; & \sum_{k=1}^{M}L_{i}\left(x_{i,k},\;u_{i,k},\;x_{i,ref}\right) & \\ \; & s.\;t. & \; & {\dot{x}}_{i}\left(t\right)-f_{i}\left(x_{i}\left(t\right),\;u_{i}\left(t\right)\right)\;=\;0\\ \; & \; & & C_{eq}\left(x_{i,k},\;u_{i,k}\right)\;=\;0\\ \; & \; & & C_{ineq}\left(x_{i,k},\;u_{i,k}\right)\;\leq \;0 \end{array}$$

The objective function implemented in this work has the following quadratic form.

(65) $$\begin{array}{cc} \; & L_{i}\left(x_{i,k},\;u_{i,k},\;x_{i,ref}\right)\;=\;\\ \; & \sum_{k=1}^{M}L_{i,tp}\left(x_{i,ref},\;x_{i,k}\right)+u_{i,k}^{T}Ru_{i,k}+x_{i,k}^{T}Qx_{i,k} \end{array}$$

where $Q\in R^{n_{x}\times n_{x}}$ and $R\in R^{n_{u}\times n_{u}}$ are square matrices. This objective function drives the system to achieve the cooperative task defined by $L_{i,tp}\left(x_{i,ref},\;x_{i,k}\right)$, which brings a nuanced cooperativeness with task prioritization to the tracking and is explained in Section 8.3.

The equality and inequality constraints serve the following purposes. $C_{eq}\left(x_{i,k},\;u_{i,k}\right)$ is introduced to ensure the initial conditions, which are $x_{i,c}\;=\;x_{i}\left(t=0\right)$ and $u_{i,c}\;=\;u_{i}\left(t=0\right)$, and $C_{ineq}\left(x_{i,k},\;u_{i,k}\right)$ is introduced to enforce states and inputs to stay in the predefined ranges. These constraints are provided in [45].

### 8.2. Linear MPC Formulation

The LMPC method implemented in this work is the implicit LMPC applied in [26,45], and details can be found therein. Therefore, the method is summarized here for completeness. The quadratic problem for the LMPC is provided in (66).

(66) $$\begin{array}{ccccc} \; & \underset{u_{i,K}}{\mathrm{minimize}} & \; & {\bar{L}}_{i}\left(x_{i,K},\;\Delta u_{i,K},\;x_{i,ref}\right) & \\ \; & \; & & {\bar{C}}_{eq}\left(x_{i,K},\;u_{i,K}\right)\;=\;0\\ \; & \; & & {\bar{C}}_{ineq}\left(x_{i,K},\;u_{i,K}\right)\;\leq \;0 \end{array}$$

The quadratic objective function is denoted as ${\bar{L}}_{i}\left(x_{i,K},\;\Delta u_{i,K},\;x_{i,ref}\right)$ and given in (67), where the decision variable is $\Delta u_{i,K}$ and $x_{i,K}$ is a function of $\Delta u_{i,K}$.

(67) $$\begin{array}{cc} \; & {\bar{L}}_{i}\left(x_{i,K},\;\Delta u_{i,K},\;x_{i,ref}\right)\;=\;\\ \; & {\bar{L}}_{i,tp}\left(x_{i,ref},\;x_{i,K}\right)+u_{i,K}^{T}\bar{R}u_{i,K}+x_{i,K}^{T}\bar{Q}x_{i,K} \end{array}$$

where $\bar{R}\;=\;diag{\left\{R\right\}}_{1}^{M}$, $\bar{Q}\;=\;diag{\left\{Q\right\}}_{1}^{M}$ and ${\bar{L}}_{i,tp}\left(x_{i,ref},\;x_{i,K}\right)$ is calculated in Section 8.3.

Due to the implicit formulation, state predictions are described with respect to the decision variables and combined in a lumped representation. Similarly, decision variables, which are the inputs, are also collected under a lumped term. These lumped states and inputs are denoted as $\Delta x_{i,K}$ and $\Delta u_{i,K}$ and provided in (68).

(68) $$\begin{array}{cc} \Delta x_{i,K}\; & =\;\left[\begin{array}{c} \Delta x_{i,k}\\ \vdots \\ \Delta x_{i,k+M-1} \end{array}\right]\\ \Delta u_{i,K}\; & =\;\left[\begin{array}{c} \Delta u_{i,k}\\ \vdots \\ \Delta u_{i,k+M-1} \end{array}\right] \end{array}$$

The relationship between $\Delta x_{i,K}$ and $\Delta u_{i,K}$ is provided below with matrices $F_{i}\in R^{Mn_{x}\times n_{x}}$ and $H_{i}\in R^{Mn_{x}\times Mn_{u}}$ for the prediction horizon of *M*.

(69) $$\begin{array}{cccc} \Delta x_{i,K} & \;=\;F_{i}x_{i,c}+H_{i}\Delta u_{i,K}\;\; & \; & \\ F_{i} & \;=\;col{\left\{F_{i,m}\right\}}_{m=1}^{M}\;\; & \; & \\ F_{i,m} & \;=\;A_{i}^{\left(m-1\right)}\;\; & ,\;m & =\left[1,\cdots ,M\right]\\ H_{i} & \;=\;col{\left\{H_{i,m}\right\}}_{m=1}^{M}\;\; & \; & \\ H_{i,m} & \;=\;0\;\; & \;m & =1\\ H_{i,m} & \;=\;row\{h_{i,l}\}\;\; & \;m & =\left[2,\cdots ,M\right]\\ h_{i,l} & \;=\;A_{i}^{\left(m-l-1\right)}B_{i}\;\; & ,\;l & =\left[1,\cdots ,m-1\right] \end{array}$$

Terminal states are given as a function of $x_{i,k}$ and $\Delta u_{i,K}$ below.

(70) $$\begin{array}{cc} \Delta x_{i,k+M} & \;=\;A_{i}^{M}x_{i,k}+{\bar{B}}_{i}\Delta u_{i,K}\\ {\bar{B}}_{i} & \;=\;\left[\begin{array}{cccc} A_{i}^{M-1}B_{i} & \cdots & A_{i}^{1}B_{i} & B_{i} \end{array}\right] \end{array}$$

${\bar{C}}_{eq}\left(x_{i,K},\;u_{i,K}\right)$ and ${\bar{C}}_{ineq}\left(x_{i,K},\;u_{i,K}\right)$ serve the same purpose with $C_{eq}\left(x_{i,k},\;u_{i,k}\right)$ and $C_{ineq}\left(x_{i,k},\;u_{i,k}\right)$; however, they are modified to comply with LMPC notation. The calculations of these constraints are provided in [26].

### 8.3. Cooperative Task Prioritization

Let $\epsilon_{i,t}\in R^{n_{\epsilon t}}$, as provided in (71), represent a cooperative task for agent *i* based on local neighbor state information that is received from neighboring agents, denoted as *j*. Assume that $\epsilon_{i,t}\in R^{n_{\epsilon t}}$ is defined for a subset of states denoted as ${\hat{x}}_{i}\subset x_{i}$ and ${\hat{x}}_{j}\subset x_{j}$, where ${\hat{x}}_{i}\in R^{n_{s}}$ and ${\hat{x}}_{j}\in R^{n_{s}}$. It should be noted that ${\hat{x}}_{i}$ and ${\hat{x}}_{j}$ are assumed to be measured with respect to the same coordinate frame. Otherwise, the task $\epsilon_{i,t}$ becomes non-linear.

(71) $$\epsilon_{i,t}\;=\;a_{ij}\left({\hat{x}}_{j}-{\hat{x}}_{i}\right),\;\;t=1,\cdots ,T$$

where *T* is the maximum number of tasks. The first derivative of $\epsilon_{i,t}$ is calculated as follows:

(72) $$\begin{array}{cc} {\dot{\epsilon }}_{i,t} & \;=\;a_{ij}\left({\dot{\hat{x}}}_{j}-{\dot{\hat{x}}}_{i}\right)\\ {\dot{\epsilon }}_{i,t} & \;=\;\left[\begin{array}{cc} a_{ij}I_{n_{s}} & -a_{ij}I_{n_{s}} \end{array}\right]\left[\begin{array}{c} {\dot{\hat{x}}}_{j}\\ {\dot{\hat{x}}}_{i} \end{array}\right] \end{array}$$

Collecting the terms on the right-hand side $M_{i}\left(a_{ij}\right)=\left[\begin{array}{cc} a_{ij}I_{n_{s}} & -a_{ij}I_{n_{s}} \end{array}\right]$ and ${\dot{S}}_{i,t}={\left[\begin{array}{cc} {\dot{\hat{x}}}_{j}^{T} & {\dot{\hat{x}}}_{i}^{T} \end{array}\right]}^{T}\in R^{n_{st}}$, the mapping in (72) between task space (TS) velocities and local state space (LSS) velocities takes the form

(73) $${\dot{\epsilon }}_{i,t}\;=\;M_{i}\left(a_{ij}\right){\dot{S}}_{i,t}$$

Following that, the inverse mapping from TS to LSS is calculated as

(74) $${\dot{S}}_{i,t}\;=\;M_{i}^{+}{\dot{\epsilon }}_{i,t}$$

where $M_{i}^{+}$ is the pseudo inverse of $M_{i,t}$ and $a_{ij}$ is dropped from the expression for brevity. Note that $x_{j}$ is the state of a neighboring agent.

As illustrated in [47] for robots, excessive (redundant) LSS can be utilized to manage secondary tasks. Let $\epsilon_{i,1}$ and $S_{i,1}$ denote the first TS and LSS and $\epsilon_{i,t}$, and let $S_{i,t},\;t=2,\cdots ,T$ denotes the remaining ones; then, following task management methodology can be used [40]:

(75) $$\begin{array}{cc} {\dot{S}}_{i,1} & \;=\;M_{i,1}^{+}{\dot{\epsilon }}_{1}\\ {\dot{S}}_{i,t} & \;=\;{\dot{S}}_{i,t-1}+{\left(M_{i,t}\Phi_{i,t-1}\right)}^{+}\left({\dot{\epsilon }}_{i,t}-M_{i,t}{\dot{S}}_{i,t-1}\right) \end{array}$$

where $\Phi_{i,t}$ is the null space projection matrix and calculated is as follows:

(76) $$\begin{array}{c} \Phi_{i,1}\;=\;I_{n_{st}}-M_{i,1}^{+}M_{i,1}\\ \Phi_{i,t}\;=\;\Phi_{i,t-1}-{\left(M_{i,t}\Phi_{i,t-1}\right)}^{+}\left(M_{i,t}\Phi_{i,t-1}\right) \end{array}$$

The formulation defined in (75) is collected in a minimal representation as in (77).

(77) $$\dot{S}\;=\;\Psi \dot{\epsilon }$$

where ${\dot{S}}_{i}\;=\;{\left[\begin{array}{cccc} {\dot{S}}_{i,1}^{T} & {\dot{S}}_{i,t}^{T} & \cdots & {\dot{S}}_{i,T}^{T} \end{array}\right]}^{T}$ and ${\dot{\epsilon }}_{i}={\left[\begin{array}{cccc} {\dot{\epsilon }}_{i,1}^{T} & {\dot{\epsilon }}_{i,t}^{T} & \cdots & {\dot{\epsilon }}_{i,T}^{T} \end{array}\right]}^{T}$. Thus, a quadratic function can be written as in (78) based on (77).

(78) $$\begin{array}{cc} L\left({\dot{\epsilon }}_{i}\right):={\dot{S}}^{T}\dot{S} & \;=\;{\dot{\epsilon }}_{i}^{T}\Psi^{T}\Psi {\dot{\epsilon }}_{i} \end{array}$$

Finally, based on (78), $L_{i,tp}\left(x_{i,ref},\;x_{i,k}\right)$ and ${\bar{L}}_{i,tp}\left(x_{i,ref},\;x_{i,K}\right)$ are calculated as given in (79) and (83), respectively, where $Q_{s}^{T}\;=\;Q_{s}\geq 0\in R^{n_{\epsilon t}\times n_{\epsilon t}}$.

(79) $$\begin{array}{cc} L_{i,tp}\left(x_{i,ref},\;x_{i,k}\right)\; & =\;{\dot{\epsilon }}_{i}^{T}\Psi^{T}\left[\begin{array}{cc} Q_{s} & 0\\ 0 & Q_{s} \end{array}\right]\Psi {\dot{\epsilon }}_{i}\\ \; & =\;{\dot{\epsilon }}_{i}^{T}Q_{\epsilon }{\dot{\epsilon }}_{i} \end{array}$$

**Remark** **1.** *If states of the agents i and j are linearly related, then ${\hat{x}}_{i}$ and ${\hat{x}}_{j}$ are linearly related; therefore $M_{i,1}$ is constant. As a result,*

- *$L({\dot{\epsilon }}_{i})$ outputs a quadratic function of ${\dot{\epsilon }}_{i,t}$ with scalars $\gamma_{t}$ such that $L\left({\dot{\epsilon }}_{i}\right)=\sum_{t=1}^{T}\gamma_{t}{\dot{\epsilon }}_{i,t}^{2}$, where $\gamma_{1}\geq \cdots \geq \gamma_{t}\geq \cdots \geq \gamma_{T}$.*
- *The relationship given for derivatives of state space and task space in* (78) *is also valid for the state space and task space as follows:*
  (80) $$\begin{array}{cc} L\left(\epsilon_{i}\right):=S^{T}S & \;=\;\epsilon_{i}^{T}\Psi^{T}\Psi \epsilon_{i} \end{array}$$
  *which results in*
  (81) $$\begin{array}{cc} L_{i,tp}\left(x_{i,ref},\;x_{i,k}\right)\; & =\;\epsilon_{i}^{T}\Psi^{T}\left[\begin{array}{cc} Q_{s} & 0\\ 0 & Q_{s} \end{array}\right]\Psi \epsilon_{i}\\ \; & =\;\epsilon_{i}^{T}Q_{\epsilon }\epsilon_{i} \end{array}$$

Let $Q_{\epsilon }$ be partitioned as in (82), and let $X_{i,ref}:=\bar{1}⊗x_{i,ref}$, where $\bar{1}={\left[1\;\cdots \;1\right]}^{T}\in R^{M}$. Then, the overall task matrix ${\bar{\epsilon }}_{i}$ can be written as an affine function of $X_{i,ref}$, and $x_{i,K})$ and ${\bar{L}}_{i,tp}\left(x_{i,ref},\;x_{i,K}\right)$ is calculated as provided in (83).

(82) $$Q_{\epsilon }\;=\;\left[\begin{array}{cc} Q_{\epsilon ,11} & Q_{\epsilon ,12}\\ Q_{\epsilon ,21} & Q_{\epsilon ,22} \end{array}\right]$$

(83) $${\bar{L}}_{i,tp}\left(x_{i,ref},\;x_{i,K}\right)\;=\;{\bar{\epsilon }}_{i}^{T}{\bar{Q}}_{\epsilon }{\bar{\epsilon }}_{i}$$

where ${\bar{Q}}_{\epsilon ,★*}\;=\;diag{\left\{Q_{\epsilon ,★*}\right\}}_{1}^{M}$ and ★,∗={1,2}.

(84) $${\bar{Q}}_{\epsilon }\;=\;\left[\begin{array}{cc} {\bar{Q}}_{\epsilon ,11} & {\bar{Q}}_{\epsilon ,12}\\ {\bar{Q}}_{\epsilon ,21} & {\bar{Q}}_{\epsilon ,22} \end{array}\right]$$

### 8.4. Implementation of the MPCs

**Figure 16:**
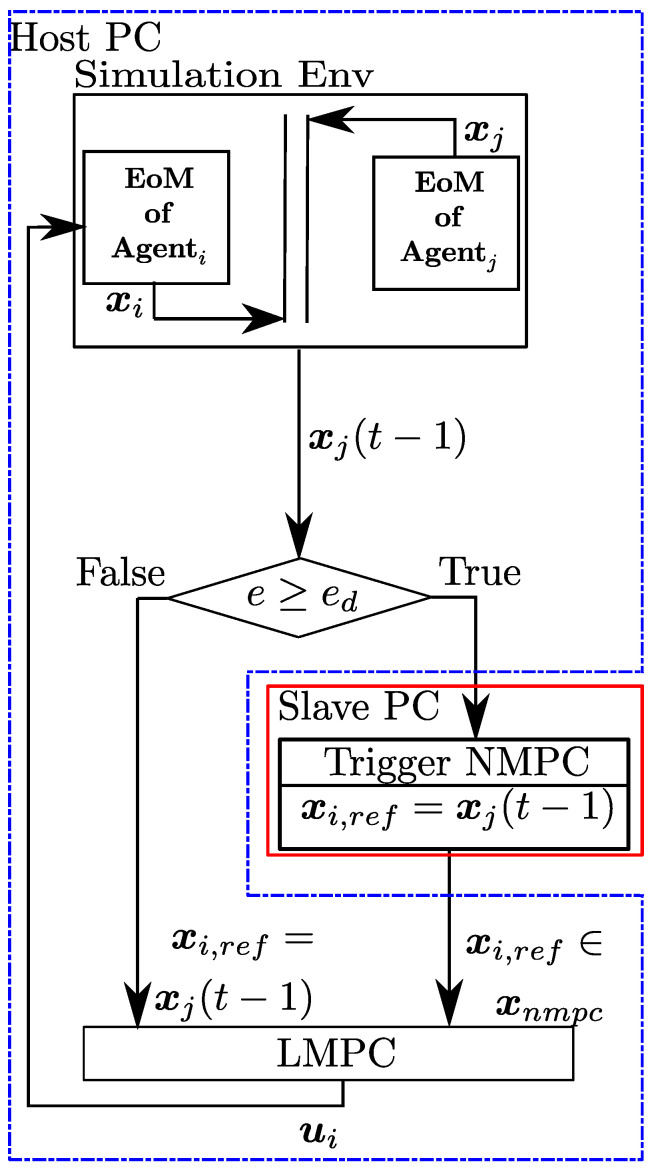
Unified MPC framework for docking.

The docking controller is designed to have several layers to decide how to perform the docking maneuver based on the distance between the agent *i* and the neighboring agents. The layers, as mentioned earlier, are the selector NMPC and LMPC. Essentially, the selector works as a governor and decides the reference trajectory that the LMPC tracks. If the absolute distance between agent *i* and neighboring agents is greater than a threshold, the NMPC generates a trajectory toward the neighboring agents using neighboring state information. Note that NMPC does not aim to achieve the terminal state constraint, but it repeatedly minimizes the state error within the given prediction horizon. Then, the generated trajectory, which creeps toward the neighbors, is passed to LMPC. If the distance is smaller than the threshold, LMPC receives the neighboring state information to generate control inputs without needing NMPC. The distance and threshold are denoted as $e\;=\;∥\left[p_{j}\right]-\left[p_{i}\right]{∥}_{2}^{2}\in R$ and $e_{d}\in R$. The currently available state and input information are denoted as $x_{i}\left(t-1\right)$, $x_{j}\left(t-1\right)$ and $u_{i}\left(t-1\right)$, where *t* represents current sample and (t−1) represents previous sample. The pipeline for the control strategy described above is given in Figure 16.

When triggered, NMPC generates a rough trajectory toward agent *j* for a given prediction horizon *M* based on the formulation given in (64). Then, a portion of this trajectory that is determined by the control horizon $M_{u}$ is extracted, and this is denoted as $x_{nmpc}$. The optimization parameters for NMPC such as *M*, $t_{f}$, and $M_{u}$ are selected empirically to achieve a rough trajectory and allow a time interval to queue a new trajectory to guide the agent *i* to the vicinity of other agents. The selection of the optimization parameters with the right-hand-side sparsity template described in [46] provides a longer time to queue a new trajectory. Before the LMPC receives the output of the NMPC or the neighboring state information, i.e., $x_{nmpc}$ ir $x_{j}\left(t-1\right)$, respectively, a line or a set of line segments are populated based on the prediction horizon of the LMPC. Finally, the tracking based on the optimization provided in (66) is performed by the LMPC.

## 9. Cooperative MPC Simulation and Results

**Figure 17:**
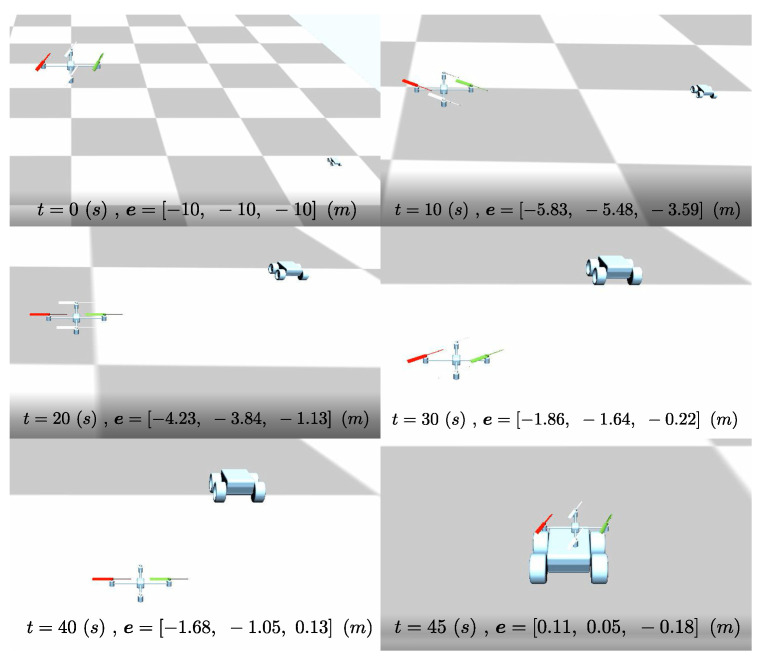
Snaps of the realized trajectory during long range docking maneuver (see [39]).

This section presents the simulation results for the proposed strategy based on the simulation setup of a quadcopter and a rover, as illustrated in Figure 17.

**Figure 18:**
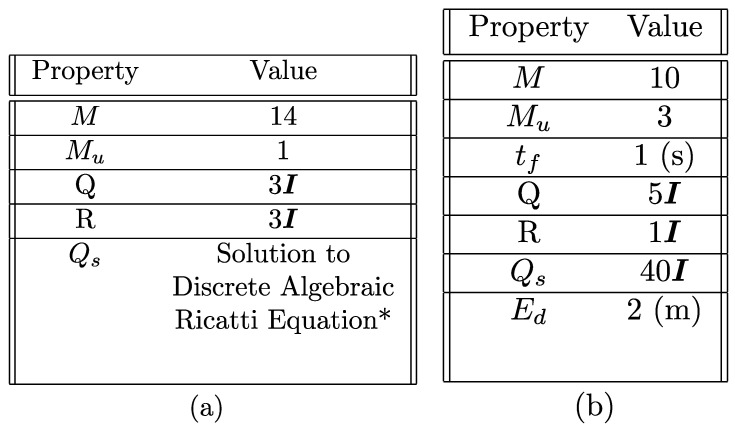
(**a**) LMPC parameters. (**b**) NMPC parameters. $t_{f}$ is the relevant time for prediction horizon. * represents the discrete algebraic Ricatti solution; see [39,48].

The NMPC and LMPC parameters are provided in Figure 18, and the quadcopter properties are given in [45] in Table 1. Two scenarios are illustrated—(1) proximity docking on a moving agent and (2) large range docking on a moving agent—where LMPC and NMPC-LMPC strategies are tested, respectively. Let $P_{s}=\left\{1,2,3,4\right\}$ represent the priority of states, where 1 is the highest priority and 4 is the lowest priority, and s=p,v,e,w represents the subset of states that P is representing. On this basis, LMPC uses the priority map of $\left\{P_{p},P_{v},P_{e},P_{w}\right\}=\left\{2,3,1,4\right\}$. Priority mapping is used to arrange tasks defined as $\epsilon_{i,t}$, where i∈{1,2,3,4}. This mapping is valid for both LMPC and NMPC.

### 9.1. Case Study 1: Proximity Docking on the Rover

The initial conditions for the quadcopter and the rover are $x_{q,0}\;=$${\left[0\;0.5\;-10\;0\;0\;0\;0\;0\;0\;0\;0\;0\right]}^{T}$ and $x_{q,0}\;=\;{\left[0\;0\;0\;0.5\;0\;0\;0\;0\;0\;0\;0\;0\right]}^{T}$, respectively.

**Figure 19:**
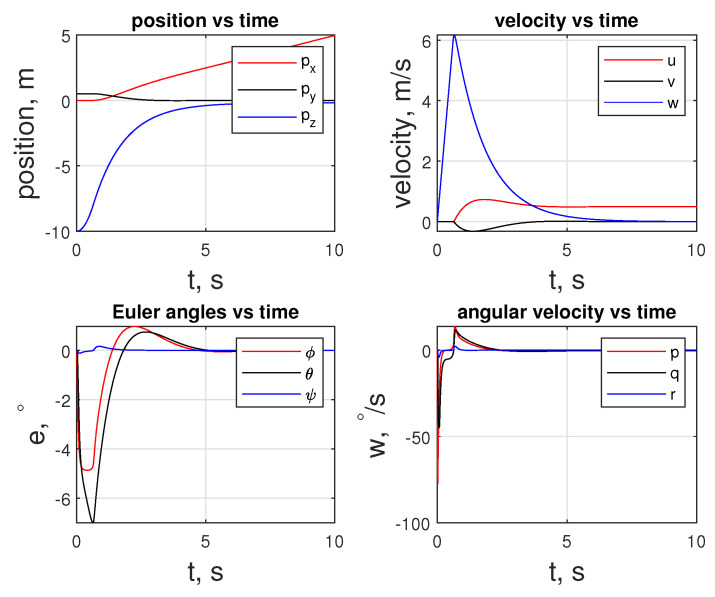
State trajectory of the agent of the quadcopter.

The initial inputs for the quadcopter are $u_{i,c}\;=\;{\left[9.81m_{q}\;0\;0\;0\right]}^{T}$ to temper the behavior of the $f_{q}$. Given the initial values, the quadcopter is guided to the rover with −0.1 m offset in the *z* direction, as illustrated in Figure 19. As shown in Figure 19, the quadcopter makes a free fall in $3/4^{th}$ of a second, and then it recovers. During this phase, it loses altitude and gains speed in the *w* component up to 6m/s. The duration of the free fall is correlated to R in the cost function and inequality constraints provided to the LMPC, which is ${\bar{C}}_{ineq}\left(x_{i,K},\;u_{i,K}\right)$. After the free fall, after approximately t=1 (s), the controller makes a correction in e and starts approaching the rover in the x- and y-directions.

**Figure 20:**
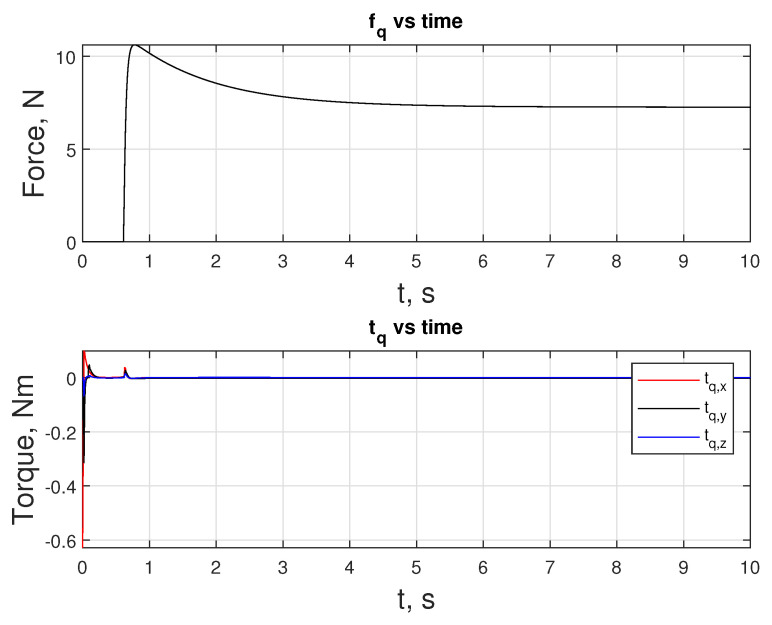
Inputs $f_{q}$ and $t_{q}$ over the duration of the docking maneuver.

As mentioned above, free fall can also be seen in Figure 20 in the $f_{q}$ subplot, where $f_{q}$ stays at 0 N for that period. During the maneuver, $f_{q}$ is upper-bounded by 12 N. The maximums for $t_{q}$ are reached in the x-direction, where −0.52 and 0.05 Nm of toque are observed at the extremes.

**Figure 21:**
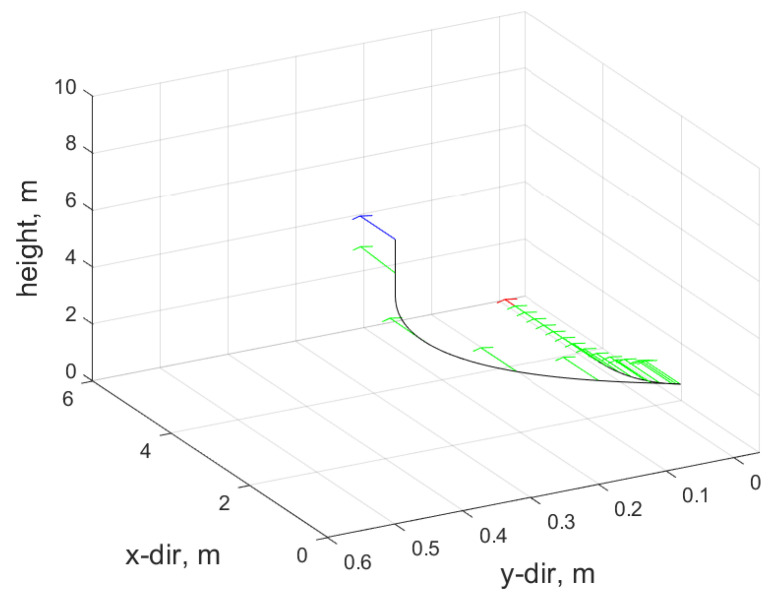
Trajectory and heading of the quadcopter.

Figure 21 provides an overview of the performed trajectory along with the heading direction of the quadcopter, which is aligned with the $u_{1}$ axis of $F_{B}$.

### 9.2. Case Study 2: Long-Range Docking on the Rover

The initial conditions for the quadcopter and the rover are $x_{q,0}\;=\;{\left[0\;0\;-10\;0\;0\;0\;0\;0\;0\;0\;0\;0\right]}^{T}$ and $x_{r,0}\;=\;{\left[10\;10\;0\;0.5\;0\;0\;0\;0\;0\;0\;0\;0\right]}^{T}$, respectively.

The initial inputs for the quadcopter are $u_{i,c}\;=\;{\left[9.81m_{q}\;0\;0\;0\right]}^{T}$. This case illustrates the long-range capability of the proposed control strategy. The overview of the scenario is provided as a set snapshot in Figure 17, and the video of the performed scenario can be found in https://www.youtube.com/watch?v=QJ-NuwZQ8zw (accessed on 12 May 2024).

**Figure 22:**
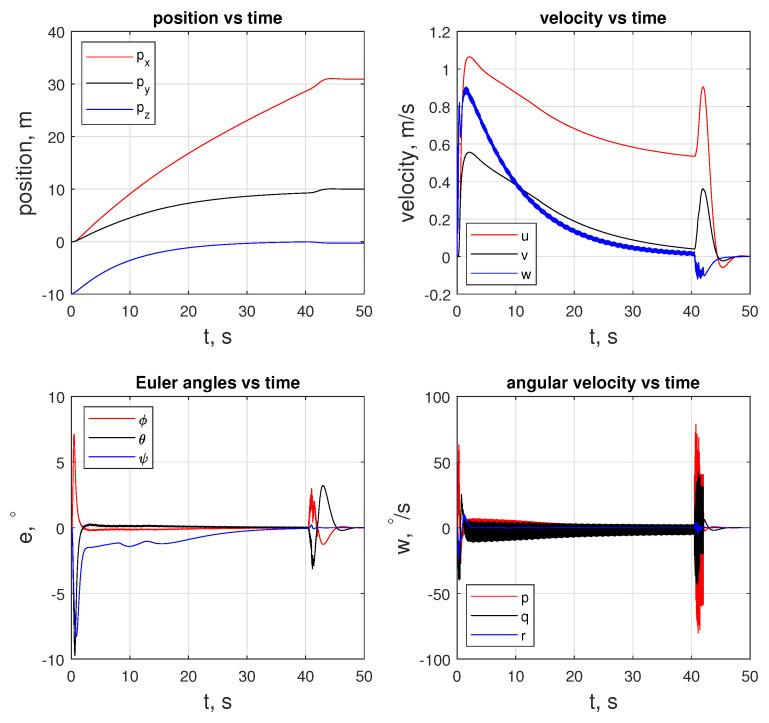
State trajectory of the agent of the quadcopter.

**Figure 23:**
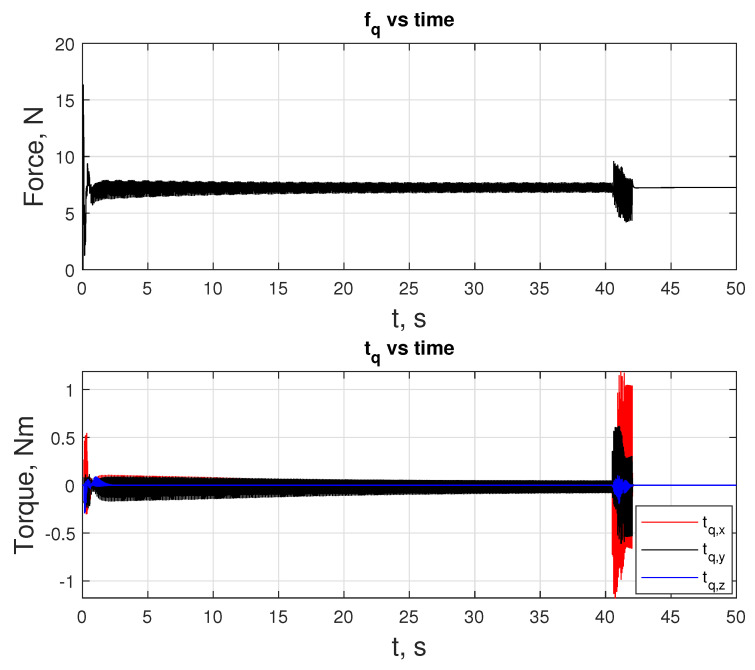
Inputs $f_{q}$ and $t_{q}$ over the duration of the docking maneuver.

**Figure 24:**
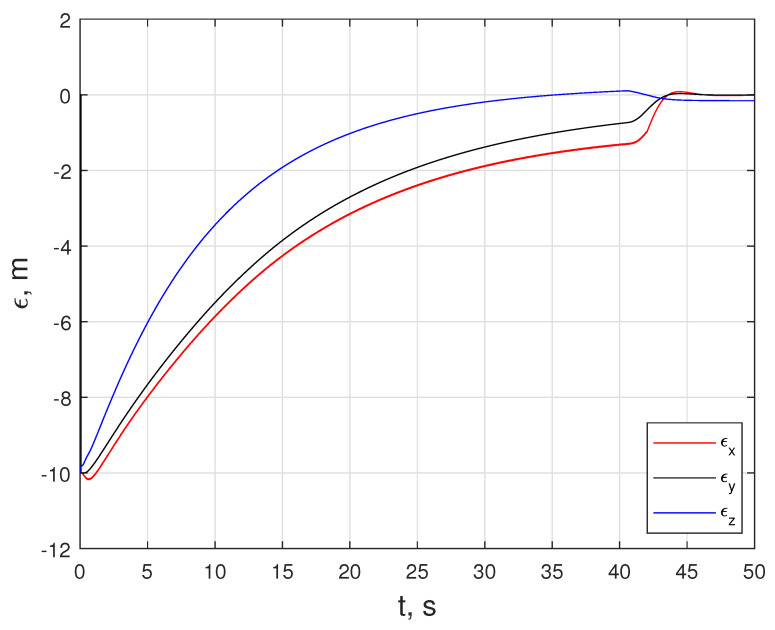
Position error between the rover and the quadcopter.

In this scenario, LMPC tracks the $x_{nmpc}$ that the NMPC generates until e is less than $e_{d}$. At about 41 s, a transition happens, and LMPC starts tracking the $x_{j}\left(t-1\right)$. Figure 22 shows the states of the quadcopter. An important feature of this motion is that the signal oscillates at a velocity level in both the linear and angular motion. The same oscillatory behavior is visible for inputs $f_{q}$ and $t_{q}$, as provided in Figure 23, which is because the quadcopter’s current states are changing as the $x_{nmpc}$ is generated on the NMPC side, sent to the LMPC, and populated as line segments. Figure 22 and Figure 24 reveal the difference in proximity and long-range docking maneuvers, to which LMPC is more sensitive and can be seen from the magnitude of the tracking error e.

The positional errors between the rover and the quadcopter in Figure 24 illustrate that the positional errors in the x− and y−directions approach zero, while the error in the z−direction approach −0.1 m.

**Figure 25:**
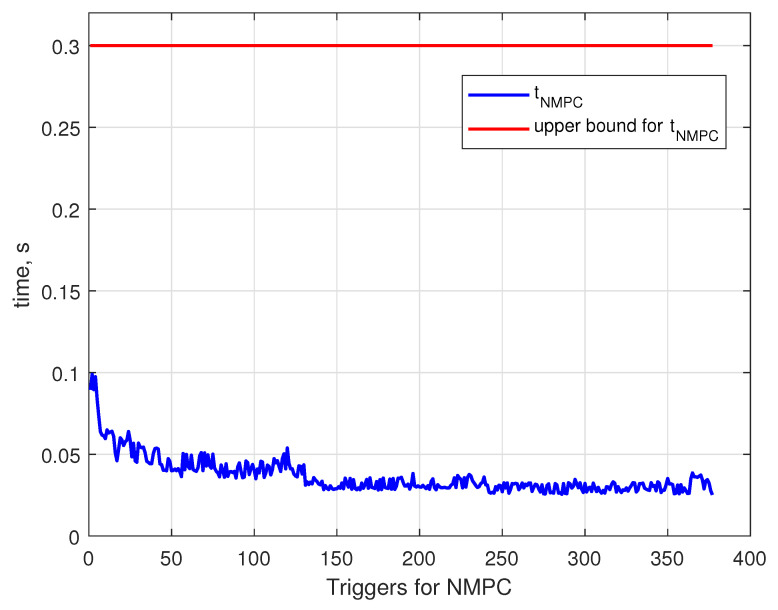
Calculation time for the NMPC problem at every instant.

Figure 25 illustrates the calculation time of each NMPC trigger. Initially, the generation costs approximately 0.1 s, and as the quadcopter settles on the trajectory trajectory generation, the times decrease to 0.05 s. For a given elapsed time profile, the overall calculation time is calculated to be 13.91 s. The upper bound line illustrates the control horizon $M_{u}$ for the NMPC; see [39]).

## 10. Discussion

The *central premise* of this paper is to show that a complex dynamical system and/or a control system can be decomposed into parts. These parts can be constrained to develop the governing equations to study the dynamics or to synthesize a control strategy for the composite system using the the interconnection between the sub-systems as constraints. Note thatwhile these are two separate applications, they can be studied using the same framework. In the biped dynamics, the legs (left and right) and the torso are considered as sub-systems, which are coupled via constraints and thus modeled using the cooperative system framework. The trajectory for the biped robot is computed using a sequence of optimizations. Given a desired amount of forward movement of the center of mass, one would begin from the contact phase optimization, the results of which then feed into the swing phase optimization, hwich provides the necessary smooth trajectories for moving to the new location. This is finally passed on to the cooperative force optimization, which computes the necessary generalized forces to accomplish the movement.

While there are promising early results from this approach, further study is warranted to investigate the sensitivity of the optimization approaches to parameters such as the number of phases, the number of sample instants, the sampling period, the contact force models (friction cone), and the asymmetry in the legs, sensor noise, and other unmodeled terms in the dynamics.

Similarly, in the case of a cooperative docking scenario, the quadcopter and ground rover are two separate sub-systems of the overall system and are constrained to work together in the cooperative setup. In this context, the framework also allows one to choose different control schemes and combine them together for the overall cooperative mission. For example, a nonlinear MPC was chosen to bring the quadcopter closer to the ground rover faster because we wish for a faster approach to the goal; however, when one is close to the goal, a linear MPC controller is activated since when it is closer to the desired state, the linear terms typically dominate higher-order polynomial terms and/or harmonic functions.

In the cooperative task decomposition for the UAV-UGV problem, one would have to further study the impact of communication delays and environmental disturbances such as unstructured terrain models to fully understand the efficacy of the proposed cooperative control formulation.

## 11. Summary and Conclusions

This paper presents a cooperative modeling framework that is shown to reduce the complexity of deriving the governing dynamical equations of complex systems composed of multiple bodies. The approach also allows for an optimization-based trajectory generation for the complex system.

The results show that such cooperative modeling enables online trajectory generation through a series of optimizations that generate approximate trajectories and pass them to a final optimization that refines the motion with the cooperative system framework.

The paper also proposes a unified MPC control strategy capable of handling long-range to proximity docking maneuvers. A rough but fast NMPC method is run to propagate the agent to a closer vicinity, and then the LMPC handles more sensitive proximity motion. The sensitivity of the LMPC is apparent in Figure 22 and Figure 24, where a smooth approach suddenly becomes relatively violent. Based on the simulations, the selection of the matrices Q, R and $Q_{s}$ makes the transition from NMPC-LMPC to LMPC smoother. Note that the docking of the quadcopter is almost tangential to the x−y plane, and during the final section of the maneuver, it penetrates the docking platform. The latter problem can be resolved with an approach constraint to the MPC controllers. Most importantly, the proposed method is readily applicable among dynamic systems, including non-linear systems, since the controller’s structure stays the same. Using local neighbor information instead of external sensors or observers naturally makes this control strategy decentralized.

## Figures and Tables

**Table 1:** Summary of key variables used for the description of the ASLB kinematics and dynamics.

$q_{B}$	set of unactuated joints
$q_{t,B}$	translational joints
$q_{r,B}$	rotational joints
$r_{B}\left(\right)$	translation matrix from one frame to another
$C_{IB}\left(\right)$	rotation matrix from frame *I* to frame *B*
$\theta_{i}$	vector of joints for each leg
$\theta_{a,i}$	vector of active joints for leg, *i*
$\theta_{p,i}$	vector of passive joints for leg, *i*
$r_{v,i}$	position of point *v*
$R_{*}$	elementary rotation matrix for * axis
$v_{r,i}$	velocity relationship
$J_{r,i}$	Jacobian relating velocity to active and passive joints
$a_{.,.}$	lengths between joints
M()	generalized mass matrix
C(.,.)	Coriolis and centrifugal terms
G()	gravitational terms
S	control selection matrix for actuated joints of respective legs
τ	actuated joint torques
$F_{C,i}$	external force on tip of $i^{\mathrm{th}}$ leg
$J_{C,i}$	geometric Jacobian of tip point for $i^{\mathrm{th}}$ leg
$\omega_{0}$	angular speed for the linear inverted pendulum model (LIPM)
x¯c,K p	contact state ${\bar{x}}_{c}$ at instant *K* in phase *p*

**Table 2:** Summary of key variables used for description of the quadcopter and rover kinematics and dynamics.

$F_{B}$	body fixed reference frame (quadcopter)
$m_{q}$	mass of the quadcopter
$J_{q}$	moment of inertia of the quadcopter
$x_{q}$	state vector of quadcopter
Cq I (θq)=Cq I	rotation matrix for quadcopter
$f_{q}$	total thrust generated by motors in the body frame
$t_{q}$	moments generated on the body
$u_{q}$	input vector for the quadcopter model
*g*	acceleration due to gravity
${\left[u_{1}\;u_{2}\;u_{3}\right]}^{T}$	unit vectors representing the body frame
$m_{r}$	mass of the rover
$J_{r}$	moment of inertia of the rover
$x_{r}$	state vector for rover dynamics
Cr I	rotation matrix of the rover
$u_{r}$	inputs to the rover

## Data Availability

All data was synthetically generated using the numerical simulation models presented in this paper.
